# Benefits of Home-Based Exercise Training Following Critical SARS-CoV-2 Infection: A Case Report

**DOI:** 10.3389/fspor.2021.791703

**Published:** 2022-01-11

**Authors:** Igor Longobardi, Danilo Marcelo Leite do Prado, Karla Fabiana Goessler, Gersiel Nascimento de Oliveira Júnior, Danieli Castro Oliveira de Andrade, Bruno Gualano, Hamilton Roschel

**Affiliations:** ^1^Applied Physiology and Nutrition Research Group, School of Physical Education and Sport, Faculdade de Medicina, Universidade de São Paulo, São Paulo, Brazil; ^2^Rheumatology Division, Hospital das Clinicas, Faculdade de Medicina, Universidade de São Paulo, São Paulo, Brazil

**Keywords:** exercise training, long COVID, fatigue, physical rehabilitation, severe acute respiratory syndrome, case report

## Abstract

In the current scenario, in which an elevated number of COVID-19 survivors present with severe physical deconditioning, exercise intolerance, persistent symptoms, and other post-acute consequences, effective rehabilitation strategies are of utmost relevance. In this study, we report for the first time the effect of home-based exercise training (HBET) in a survivor patient from critical COVID-19 illness. A 67-year-old woman who had critical COVID-19 disease [71 days of hospitalization, of which 49 days were in the intensive care unit (ICU) with invasive mechanical ventilation due to respiratory failure] underwent a 10-week HBET aiming to recovering overall physical condition. Before and after the intervention, we assessed cardiopulmonary parameters, skeletal muscle strength and functionality, fatigue severity, and self-reported persistent symptoms. At baseline (3 months after discharge), she presented with severe impairment in cardiorespiratory functional capacity (<50% age predicted VO_2peak_). After the intervention, remarkable improvements in VO_2peak_ (from 10.61 to 15.48 mL·kg^−1^·min^−1^, Δ: 45.9%), oxygen uptake efficiency slope (OUES; from 1.0 to 1.3 L·min^−1^, Δ: 30.1%), HR/VO_2_ slope (from 92 to 52 bpm·L^−1^, Δ: −43.5%), the lowest VE/VCO_2_ ratio (from 35.4 to 32.9 L·min^−1^, Δ: −7.1%), and exertional dyspnea were observed. In addition, handgrip strength (from 22 to 27 kg, Δ: 22.7%), 30-s Sit-to-Stand (30-STS; from 14 to 16 repetitions, Δ:14.3%), Timed-Up-and-Go (TUG; from 8.25 to 7.01 s, Δ: −15%) performance and post-COVID functional status (PCFS) score (from 4 to 2) were also improved from baseline to post-intervention. Self-reported persistent symptoms were also improved, and Fatigue Severity Scale (FSS) score decreased (from 4 to 2.7) from baseline to post-intervention. This is the first evidence that a semi-supervised, HBET program may be safe and potentially effective in improving cardiorespiratory and physical functionality in COVID-19 survivors. Controlled studies are warranted to confirm these findings.

## Introduction

As of December, 2021, there are over 275 million confirmed cases of coronavirus disease (COVID-19) worldwide (World Health Organization., 2021). It is estimated that nearly 20% of the infected patients develop severe cases (Wu and McGoogan, 2020), usually requiring hospitalization or even intensive care unit (ICU) support, which may last for several days or weeks (Liu et al., 2020). The combination of disease pathophysiology, treatment-related drugs' toxicity, and prolonged bed rest may result in extreme deconditioning and exercise intolerance observed at hospital discharge and months after it (Baratto et al., 2021; Raman et al., 2021). Additionally, impaired cardiorespiratory functional capacity (Blair et al., 1989; Harber et al., 2017) and skeletal muscle strength and functionality (Katzmarzyk and Craig, 2002; Leong et al., 2015; Soysal et al., 2021) are associated with higher incidence of morbidity and all-cause mortality after hospitalization.

Therefore, despite the reasonable concerns regarding the acute phase of COVID-19 infection, attention must also be directed toward survivors' rehabilitation. The benefits of exercise training are widely known and it has been considered as a first-line therapy in a variety of diseases and conditions (Pedersen and Saltin, 2015). In the context of the pandemic, home-based exercise training (HBET) programs have been strongly encouraged as this modality has been shown to improve health-related parameters and fitness in several conditions (Hong et al., 2017; Wuytack et al., 2018; Sieczkowska et al., 2021). Nevertheless, evidence regarding the benefits of exercise training in COVID-19 survivors is still lacking. Herein, we report for the first time on the effects of HBET in a critical COVID-19 survivor.

## Case Description

### Patient Information

The patient was a 67-year-old, low-income, black, Brazilian woman. In addition to SARS-CoV-2 infection (confirmed by RT-PCR for SARS-CoV-2 from nasopharyngeal swab), she had essential hypertension and hypothyroidism. The patient self-reported to be sedentary and a former smoker (tobacco load: 5 pack-year for 10-years; 25-years without smoking). Her daily-use medications included hydrochlorothiazide, losartan, levothyroxine, and clonazepam, which were kept constant in the previous months and throughout the study period.

In August 2020, the patient was admitted at an emergency care unit presenting with oxygen saturation ~70% at rest on room air and bilateral multifocal ground-glass opacities >50% assessed by lung computed tomography scan. Due to the severe and refractory hypoxemic insufficiency, she was immediately transferred to the Clinical Hospital of the School of Medicine of the University of Sao Paulo, a quaternary referral hospital. Her condition rapidly progressed to an acute pulmonary respiratory failure requiring ICU admission and intubation. After 1 week, she developed renal dysfunction (without dialysis requirement) and pulmonary thromboembolism. Within the third week of hospitalization, her clinical condition continued to deteriorate, progressing to cardiopulmonary arrest. After successful resuscitation, the patient was stabilized and began to improve slowly. However, after a sequence of unsuccessful extubating attempts, the clinical decision was for a tracheostomy. Decannulation occurred only 20 days later, and 2 weeks before her hospital discharge. In total, the patient had been hospitalized for 71 days; 49 days within the ICU, in which she remained under invasive mechanical ventilation for 47 days and received a variety of medications during hospital stay (Supplementary Material 1, Supplementary Table 1). Due to residual pneumopathy, she had been prescribed with home nocturnal oxygen therapy for the first 30 days after hospital discharge.

### Procedures

This study was approved by the Clinical Hospital of the School of Medicine of the University of Sao Paulo Ethical Committee (CAEE: 31303720.7.0000.0068). The patient provided written informed consent to participate in this study and for the publication of this case report (including any potentially identifiable images or data presented in this manuscript).

Twelve weeks after being discharged, the patient was carefully evaluated by a physician from the research team. No contraindications for exercise were found (Supplementary Material 2). Patient was not experienced with exercising before hospitalization, and she did not undergo physiotherapy or other forms of rehabilitation after hospital discharge. Assessments were performed before and after 10 weeks of intervention by the same staff member.

### Home-Based Exercise Training Program

The intervention included a 10-week semi-supervised HBET program aiming to improve her physical fitness and conditioning. It comprised three weekly sessions including aerobic, strengthening, and flexibility exercises. One weekly session was supervised by a trained researcher through live videoconference calls. The remaining two weekly sessions were monitored through text and/or voice messaging. The same researcher contacted the patient on unsupervised training days both prior to and after the scheduled hours to register adherence. In case of non-compliance, the training session was rescheduled for another day of the same week (respecting the 48-h interval between sessions). The first four training sessions were directly supervised by the same researcher through live videoconference, aiming patient's familiarization to the training protocol. In addition, supplementary materials containing exercise cards and videos, and educative information about how to rate her effort were provided (Supplementary Material 3). She was also advised to be aware of eventual symptoms (e.g., chest pain, dizziness, or others) and to immediately communicate the research team upon any symptoms for proper guidance. Oxygen saturation was not monitored throughout intervention.

Aerobic training sessions initially consisted of two bouts of 10-min/day of walk at “very light” to “fairly light” (9–11 Borg Scale) intensity (Supplementary Material 4, Supplementary Table 2). Over the following weeks, she gradually progressed toward a single 45-min bout of walking at “somewhat hard” to “hard” (14–16 Borg Scale) intensity. Additionally, she was advised to freely walk on non-training days.

Strengthening exercises comprised exercises for the major muscle groups. Exercise selection was based on baseline post-COVID functional status (PCFS) score, which was reassessed every 2 weeks as a training progression tool. A set of six strengthening exercises was designed according to each PCFS grade (Supplementary Material 5, Supplementary Table 3). She was carefully advised on how to use common household materials (i.e., water bottle, groceries packages, chair, bucket, and others) as exercise implements. Strengthening training sessions comprised 3–4 sets per exercise of 10–15 repetitions, and a self-suggested recovery interval between sets was adopted. Strength training intensity was also based on her perceived exertion and was initially set as “very light” to “fairly light” (9–11 Borg Scale) intensity and progressed toward a “somewhat hard” to “hard” (14–16 Borg Scale) intensity over the weeks of intervention (Supplementary Material 4, Supplementary Table 2). Active stretching exercises for the major muscle groups were prescribed as a cool-down.

### Cardiopulmonary Exercise Test

A maximal graded exercise test (Cardiopulmonary Exercise Test, CPET) was carried out on treadmill (Centurion model 300; Micromed, Brazil) using a modified Balke protocol (American Thoracic Society American College of Chest Physicians., 2003) at a controlled room temperature (21–23°C). Peak oxygen consumption (VO_2peak_), oxygen consumption at ventilatory anaerobic threshold (VO_2VAT_), oxygen uptake efficiency slope (OUES), oxygen pulse (VO_2_/HR), heart rate-oxygen consumption relationship (HR/VO_2_ slope), ventilatory equivalent (VE), ventilatory reserve (VE/MVV), and the lowest VE/VCO_2_ ratio were measured breath-by-breath through a computerized system (MetaLyzer 3B; Cortex, Germany) (American Thoracic Society American College of Chest Physicians., 2003; Ramos et al., 2013). Endurance time (i.e., total duration of CPET) and isotime exertional dyspnea (i.e., rate of perceived exertion at the same workload between pre- and post-intervention CPET) were also assessed.

### Muscle Strength and Functionality Assessment

Strength performance and functionality were assessed through handgrip test, 30-s Sit-to-Stand test (30-STS), and Timed-Up-and-Go test (TUG) as previously described (Balogun et al., 1991; Podsiadlo and Richardson, 1991; Jones et al., 1999). Post-COVID functional status was evaluated through specific scale (for details, see Supplementary Material 6).

### Persistent Symptoms and Fatigue Severity Assessment

Self-reported persistent symptoms were assessed through structured anamnesis encompassing a checklist of symptoms recalling since the COVID-19 symptoms onset. Fatigue severity was evaluated through a specific scale (for details, see Supplementary Material 6).

### Laboratory Assessments

Blood samples were collected after a 12-h fast and analyzed for complete blood count, lipid profile, glucose metabolism, skeletal muscle damage, cardiac muscle damage, and systemic inflammation.

## Results

### Cardiopulmonary Exercise Test

**Figure 1:**
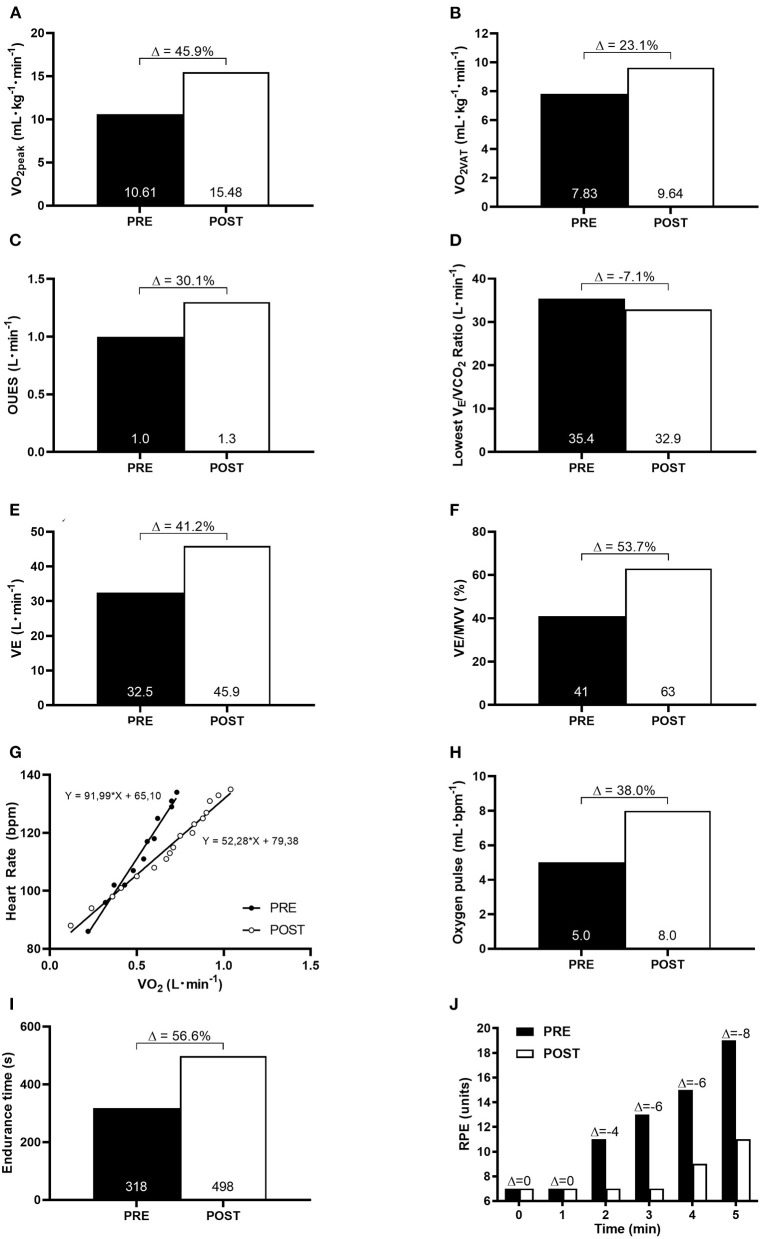
Cardiorespiratory functional capacity at baseline (PRE) and after 10-week of HBET (POST). **(A)** peak oxygen consumption (VO_2peak_); **(B)** oxygen consumption at ventilatory anaerobic threshold (VO_2VAT_); **(C)** oxygen uptake efficiency slope (OUES); **(D)** the lowest V_E_/VCO_2_ ratio; **(E)** ventilatory equivalente (VE); **(F)** ventilatory reserve (VE/MVV); **(G)** heart rate-oxygen consumption relationship (HR/VO_2_ slope); **(H)** oxygen pulse; **(I)** endurance time; **(J)** isotime comparison of rate of perceived exertion (RPE) during incremental cardiopulmonary exercise test.

At baseline, the patient presented with a severe impairment in cardiorespiratory functional capacity (<50% age predicted VO_2peak_). After 10 weeks of HBET, the patient showed remarkable improvements in aerobic fitness and oxygen uptake efficiency. VO_2peak_ increased from 10.61 to 15.48 ml·kg^−1^·min^−1^ (Δ: 45.9%), VO_2VAT_ increased from 7.83 to 9.64 ml·kg^−1^·min^−1^ (Δ: 23.1%), OUES increased from 1.0 to 1.3 L·min^−1^ (Δ: 30.1%), lowest VE/VCO_2_ ratio decreased from 35.4 to 32.9 L·min^−1^ (Δ:−7.1%), VE increased from 32.5 to 45.9 L·min^−1^ (Δ: 41.2%), ventilatory reserve (VE/MVV) increased from 41 to 63% (Δ: 53.7%), HR/VO_2_ decreased from 92 to 52 bpm·L^−1^ (Δ:−43.5%), oxygen pulse (VO_2_/HR) increased from 5 to 8 ml·bpm^−1^ (Δ: 38.0%), endurance time increased from 330 to 510 s (Δ: 56.6%), and isotime exertional dyspnea was reduced (Figures 1A–J).

### Muscle Strength and Functionality Assessments

**Figure 2:**
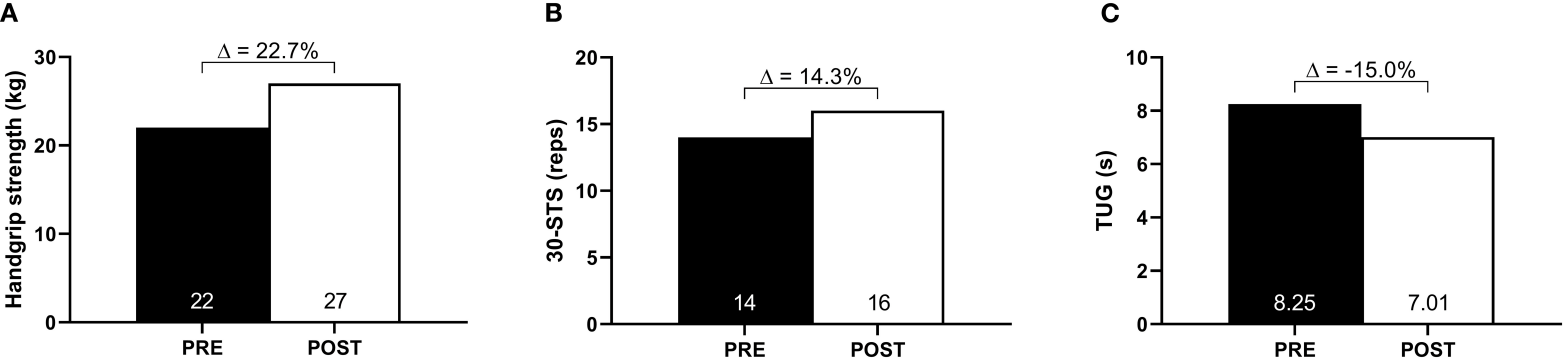
Muscle strength and functionality at baseline (PRE) and after 10-week of HBET (POST). **(A)** Handgrip strength; **(B)** 30-s sit-to-stand (30-STS); **(C)** timed-up-and-go (TUG).

Handgrip strength increased from 22 to 27 kg (Δ: 22.7%), 30-STS performance increased from 14 to 16 repetitions (Δ: 14.3%), and TUG performance improved from 8.25 to 7.01 s (Δ:−15.0%) (Figures 2A–C). Similarly, PCFS improved from 4 (severe functional impairment) to 2 (slight functional impairment).

### Persistent Symptoms and Fatigue Severity Assessments

**Figure 3:**
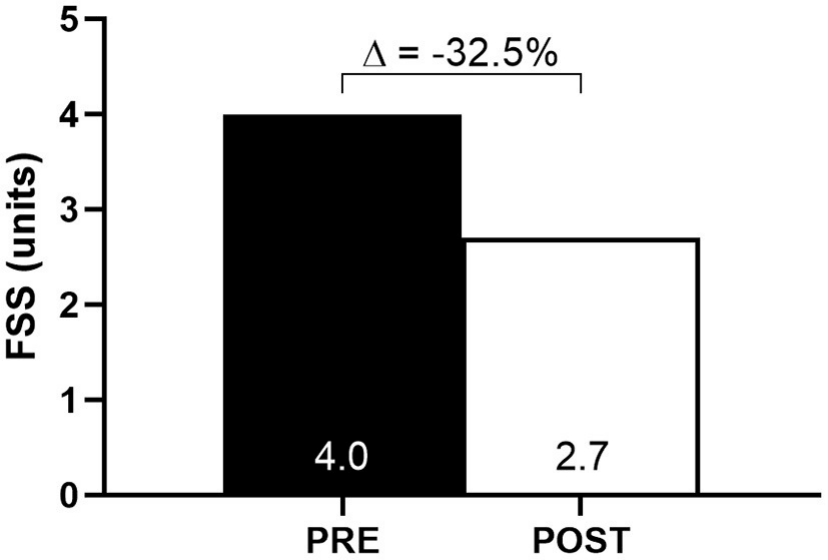
Fatigue severity scale (FSS) score at baseline (PRE) and after 10-week of HBET (POST).

At baseline, self-reported persistent symptoms included fatigue, breathlessness, weakness, myalgia, joint pain, paresthesia, dizziness, anxiety, and depression (Table 1). Only anxiety remained after the intervention. In addition, fatigue severity decreased from baseline to follow-up (pre-intervention: 4.0 vs. post-intervention: 2.7) (Figure 3).

**Table 1:** Patient's physical, clinical, and laboratorial parameters.

	**T1**	**T2**	**T3**
**Anthropometric measurements**			
BMI, kg/m^2^	27.1	28.0	28.0
Height, cm	157	-	-
Weight, kg	66.0	69.0	69.1
**Clinical measurements**			
Systolic blood pressure, mmHg	146	135	130
Diastolic blood pressure, mmHg	90	90	87
Mean arterial pressure, mmHg	109	105	101
Heart rate, bpm	98	89	85
SpO_2_, %	94	99	99
**Biochemical markers**			
Erythrocytes, 10^12^/L	3.91	4.56	4.67
Hematocrit, %	36.4	41.7	42.9
Hemoglobin concentration, g/dl	12.7	14.4	14.7
MCV, fl	94.8	92.1	91.9
MCHC, g/dl	34.9	34.3	34.3
RDW, %	13.0	14.3	12.5
White blood cell count, × 10^9^/L	7.09	6.09	6.29
Neutrophil count, × 10^9^/L	2.69	3.27	3.01
Lymphocyte count, × 10^9^/L	3.64	2.18	2.43
Monocyte count, × 10^9^/L	0.49	0.40	0.54
Platelet count, × 10^9^/L	466	379	375
Blood glucose, mmol/L	5.7	6.3	5.3
HbA1c, %	NA	6.2	5.9
Total cholesterol, mg/dl	NA	150	126
HDL-c, mg/dl	NA	48	44
LDL-c, mg/dl	NA	74	61
Triglycerides, mg/dl	NA	185	131
Creatine phosphokinase, U/L	NA	44	67
Troponin-T, pg/ml	NA	8	6
C-reactive protein, mg/L	1.6	5.9	1.8
**Persistent symptoms**			
Fatigue	–	Yes	No
Breathlessness	–	Yes	No
Weakness	–	Yes	No
Myalgia	–	Yes	No
Joint pain	–	Yes	No
Paresthesia	–	Yes	No
Dizziness	–	Yes	No
Anxiety	–	Yes	Yes
Depression	–	Yes	No

*BMI, body mass index; HbA1c, glycated hemoglobin; HDL-c, high density lipoprotein cholesterol; LDL-c, low density lipoprotein cholesterol; MCHC, mean cell hemoglobin concentration; MCV, mean corpuscular volume; NA, not available; RDW, red blood cell distribution width, SpO_2_, peripheral oxygen saturation; T1, at hospital discharge; T2: at baseline (pre-intervention), T3, post-intervention*.

### Laboratory Assessments

Hemogram was within normal limits at all times, except for the platelets that were slightly increased at hospital discharge. Lipid profile, glucose metabolism, and systemic inflammation biomarkers were slightly altered at baseline; all were within normal limits after the intervention (Table 1).

### Adherence and Adverse Events

The patient completed 29 of the 30 scheduled training sessions (i.e., adherence rate: 97%). There were no adverse events potentially associated with the intervention.

## Discussion

To the best of our knowledge, this is the first report showing that exercise training may confer clinical benefits in a survivor patient from critical COVID-19 illness. Our data showed that an individualized HBET program was safe and associated with improvements in cardiorespiratory functional capacity, skeletal muscle strength and functionality, fatigue, and most of the persistent symptoms. These findings point to the potential utility of exercise as an adjuvant therapeutic tool in the recovery of post-COVID patients, which needs to be confirmed by further controlled trials.

The patient showed a severe impairment in cardiorespiratory functional capacity at baseline, which was paralleled by physical exercise intolerance and exertion dyspnea. Our results are in accordance with previous studies which demonstrated severe deconditioning at hospital discharge and months after it (Baratto et al., 2021; Raman et al., 2021). In fact, hospitalized COVID-19 patients may experience severe deconditioning due to disease pathophysiology, treatment-related drugs' toxicity, and prolonged bed rest. Such impairment could be associated with both central (i.e., cardiac and pulmonary) and peripheral factors (i.e., skeletal muscle oxidative capacity) (Saltin et al., 1968; McGuire et al., 2001; Dirks et al., 2016; Ohtake et al., 2018). Unpublished data from our group also indicate substantial muscle atrophy in COVID-19 patients, which is probably accompanied by reduced oxidative metabolism capacity. Furthermore, the patient showed lower ventilatory efficiency as observed by an increase in lowest VE/VCO_2_ ratio and hyperkinetic circulatory response to exercise as noted by steeper HR/VO_2_ slope. Additionally, our data demonstrated lower values for OUES suggesting an impairment in oxygen uptake efficiency during exercise.

The most striking finding of the present case study was the potential benefits associated with the HBET program in improving both cardiorespiratory functional capacity and ventilatory efficiency in a survivor of critical COVID-19 illness. The remarkable improvements in HR/VO_2_ slope and oxygen pulse observed in comparison to baseline indicates a decrease in tachycardia for a given oxygen consumption. Moreover, we observed an expressive change in VO_2peak_, greater than values commonly seen in other populations of chronic diseases, such as in heart failure and chronic obstructive pulmonary disease (Tabet et al., 2009; Puente-Maestu et al., 2016). Importantly, these patients show structural and functional abnormalities that can further limit exercise training progression (and, thus, its benefits) and CPET performance as compared to our COVID-19 survivor. It is imperative to consider, however, that ours is a single case of an emblematic patient that, in addition to the exercise training, was still recovering from an acute phase of a critical respiratory infection. Owning to the fact that our patient's cardiorespiratory functional capacity was still severely compromised at baseline, greater improvements in cardiorespiratory variables were expected to occur in response to exercise training due to the low initial level of aerobic fitness (Prado et al., 2015). Nonetheless, collectively, these findings suggest an increase in both stroke volume and/or oxygen extraction by peripheral tissues (Ramos et al., 2013; Mitchell et al., 2019).

Functionality is closely related to independence, performance of activities of daily living, and quality of life (Guralnik et al., 1995; Shinkai et al., 2000; Onder et al., 2005). Post-COVID functional status score at the baseline indicated severe functional limitations. Interestingly, all functional tests scores were within the expected range for the patient's age and sex despite the disease, suggesting that the observed physical limitations may be mostly related to cardiorespiratory deconditioning. Nevertheless, in addition to the improvements in both oxygen uptake capacity and dyspnea, the enhancement in lower-limb strength and functionality may have also contributed to a reduction in PCFS score following the intervention.

Fatigue is one of the most limiting symptoms and the most common persistent symptom reported by COVID-19 survivors (Carfi et al., 2020), which may last for several months after disease onset (Huang et al., 2021). Importantly, fatigue and all of the other persistent symptoms, except anxiety, were resolved after the intervention.

The strengths of this study involve the longitudinal assessment of exercise as a novel adjuvant therapeutic tool in a survivor of critical COVID-19 illness, and the use of valid and comprehensive measures of physical capacity and functionality. The major limitation is inherent to the nature of the study, which provides novel insights into the potential role of exercise in post-COVID-19 but does not allow firm conclusions on safety and efficacy of the intervention in this condition, since only a single patient was examined. It cannot be ruled out that the changes across time reported in this study may be resultant from the natural course of the disease and/or individual factors that have not been assessed.

In summary, this study provides preliminary evidence that an exercise training program may be safe and potentially effective in recovering cardiorespiratory functional capacity, functionality, fatigue, exertional dyspnea, and other persistent symptoms in COVID-19 survivors. While caution should be exercised in interpreting the present findings in light of the limitations inherent to a case study, the data reported herein are encouraging and may help pave the way for randomized controlled trials testing the safety, efficacy and feasibility of exercise interventions as an adjuvant therapeutic tool in post-COVID-19 syndrome.

## Data Availability Statement

The original data presented in the study are included in the article/Supplementary Material, further inquiries can be directed to the corresponding author.

## Ethics Statement

The studies involving human participants were reviewed and approved by Clinical Hospital of the School of Medicine of the University of Sao Paulo Ethical Committee. Written informed consent was obtained from the individual for the publication of this case report (including any potentially identifiable images or data presented in this manuscript).

## Author Contributions

IL participated in the design of the study, contributed to data collection, data analysis, and contributed to interpretation of results. DP contributed to data collection, data analysis, and interpretation of results. KG and DA participated in the design of the study. GO contributed to data collection. BG and HR participated in the design of the study and contributed to interpretation of results. All authors contributed to the manuscript writing, read, and approved the final version of the manuscript and agreed with the order of presentation of the authors.

## Funding

IL has been financially supported by CAPES (#88887.624726/2021-00). KG, GO, and BG has been FAPESP (#2019-18039-7, #2020/07540-4, and #2017-13552-2). HR has been financially supported by CNPq (#301571/2017-1).

## Conflict of Interest

The authors declare that the research was conducted in the absence of any commercial or financial relationships that could be construed as a potential conflict of interest.

## Publisher's Note

All claims expressed in this article are solely those of the authors and do not necessarily represent those of their affiliated organizations, or those of the publisher, the editors and the reviewers. Any product that may be evaluated in this article, or claim that may be made by its manufacturer, is not guaranteed or endorsed by the publisher.

## Supplementary Material

Supplementary Material 1 Supplementary Material 1 is available at https://figshare.com/s/70c450f8f5423aef2197.

Supplementary Material 2 Supplementary Material 2 is available at https://figshare.com/s/bea1c4d82c31f4c62ca7.

Supplementary Material 3 Supplementary Material 3 is available at https://figshare.com/s/f0bc4ae01bde1097bb3a.

Supplementary Material 4 Supplementary Material 4 is available at https://figshare.com/s/30f348a90e8d4faad445.

Supplementary Material 5 Supplementary Material 5 is available at https://figshare.com/s/6d3dcda8da82a12c43c2.

Supplementary Material 6 Supplementary Material 6 is available at https://figshare.com/s/d91371b8c3fe90c7b732.
