# Supplementary material for: Benefits of Home-Based Exercise Training Following Critical SARS-CoV-2 Infection: A Case Report
Source: Front Sports Act Living. 2022 Jan 11;3:791703. doi: 10.3389/fspor.2021.791703 (PMC8787158; doi:10.3389/fspor.2021.791703)
Supplement: Supplementary Material 1 — is available at https://figshare.com/s/70c450f8f5423aef2197. [file Data_Sheet_1.PDF]

Supplementary Material 1

Supplementary Table 1. Drug therapy during hospital stay.

Drug class	Drugs administered
Antibiotics	• Ceftriaxone<br>• Piperacillin<br>• Tazobactam<br>• Vancomycin<br>• Oxacillin
Bronchodilators	• Salbutamol Sulfate
Corticosteroids	• Dexamethasone<br>• Prednisolone<br>• Hydrocortisone<br>• Prednisone
Nonsteroidal anti-inflammatory	• Metamizole
Sedatives	• Midazolam<br>• Propofol<br>• Dexmedetomidine<br>• Etomidate
Analgesics	• Fentanyl<br>• Morphine<br>• Methadone<br>• Dextroketamine<br>• Tramadol
Neuromuscular blockers	• Rocuronium<br>• Cisatracurium
Anticoagulants	• Enoxaparin<br>• Warfarin
Antihypertensive	• Losartan<br>• Atenolol<br>• Amlodipine<br>• Clonidine<br>• Hydrochlorothiazide<br>• Furosemide
Parasympatholytic	• Atropine
Vasoconstrictors	• Norepinephrine<br>• Vasopressin
Ophthalmic drugs	• Timolol

	• Hydroxypropyl Methylcellulose
Antipsychotic/antidepressant	• Quetiapine (Fumarate)<br>• Clonazepam
Antidiabetic	• Insulin
Thyroid therapy	• Levothyroxine
Proton pump inhibitors	• Omeprazole
Laxatives	• Lactulose<br>• Glycerin<br>• Bisacodyl
Anti-flatulence	• Simethicone
