# Supplementary material for: Benefits of Home-Based Exercise Training Following Critical SARS-CoV-2 Infection: A Case Report
Source: Front Sports Act Living. 2022 Jan 11;3:791703. doi: 10.3389/fspor.2021.791703 (PMC8787158; doi:10.3389/fspor.2021.791703)
Supplement: Supplementary Material 2 — is available at https://figshare.com/s/bea1c4d82c31f4c62ca7. [file Data_Sheet_2.PDF]

Supplementary Material 2

***<u>Possible conditions assessed during the pre-participation screening for home-based exercise training eligibility</u>*:**

1) recent myocardial infarction (<12 months);

2) complete heart block, third-degree atrioventricular block or advanced atrioventricular block;

3) atrial fibrillation;

4) complex ventricular arrhythmias;

5) congestive heart failure;

6) cardiac thrombosis;

7) unstable angina;

8) dyspnea at rest;

9) severe valve disease;

10) cardiomyopathy;

11) acute myocarditis or pericarditis;

12) pulmonary hypertension;

13) acute pulmonary embolism or pulmonary infarction;

14) deep venous thromboembolism;

15) uncontrolled visual or vestibular disorders;

16) uncontrolled resting tachycardia;

17) uncontrolled hypertension;

18) uncontrolled type II diabetes;

19) acute infections;

20) history of recent malignancy (<5 years);

21) transplant history;

22) autoimmune diseases;

23) dementia that disables the follow-up of the intervention;

24) any physical limitation that prevents the performance of exercise tests or physical exercise training.
