# Supplementary material for: Benefits of Home-Based Exercise Training Following Critical SARS-CoV-2 Infection: A Case Report
Source: Front Sports Act Living. 2022 Jan 11;3:791703. doi: 10.3389/fspor.2021.791703 (PMC8787158; doi:10.3389/fspor.2021.791703)
Supplement: Supplementary Material 3 — is available at https://figshare.com/s/f0bc4ae01bde1097bb3a. [file Data_Sheet_3.PDF]

RATE YOUR PERCEIVED EXERTION RIGHT AFTER EACH EXERCISE & AFTER THE TRAINING SESSION

1) Identifying your RPE

How intense was your walk?

Ex: "I didn't get tired at all, it was **very light**, thus, my RPE is **10**!"

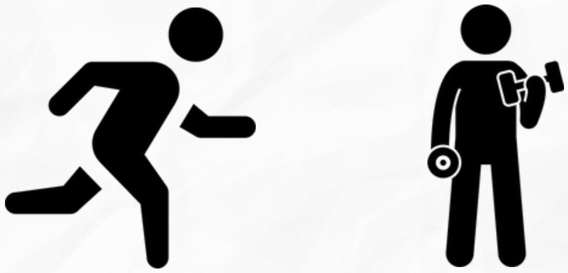

How intense was the strengthening exercise that you've just performed?

Ex: "It was **hard** to perform knee extensions, thus, my RPE is **15**!"

TAKE THESE ASSESSMENTS RIGHT AFTER **EACH EXERCISE**!

Rate of perceived exertion (RPE)

Borg's RPE Scale

RPE	Description
6, 7, 8	Very, very light
9, 10	Very light
11, 12	Fairly light
13, 14	Somewhat hard
15, 16	Hard
17, 18	Very hard
19, 20	Very, very hard

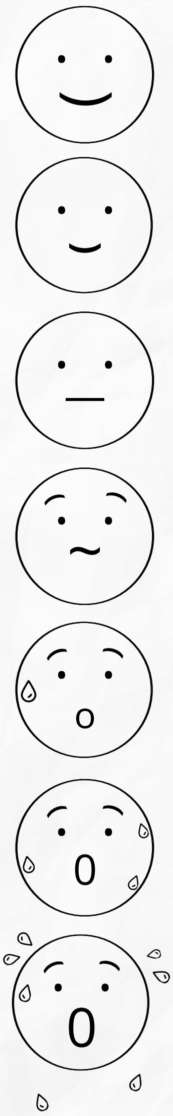

2) Identify your exertion after the training session

TRAINING SESSION =

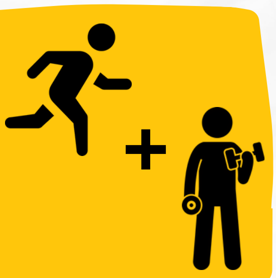

30 MINUTES after completing all exercises (both aerobic and strengthening), rate the general RPE of the training session.

Think on how intense/heavy/difficult the **whole training session** was and not just a single exercise.

Ex: "Overall, today's session was **somewhat hard**, so my session RPE is **13**!"

Provide this information to the researcher who is training you immediately after identifying the respective RPEs

EXERCISE CARDS

PCFS grade: 4

WARM-UP

WRIST ROTATIONS

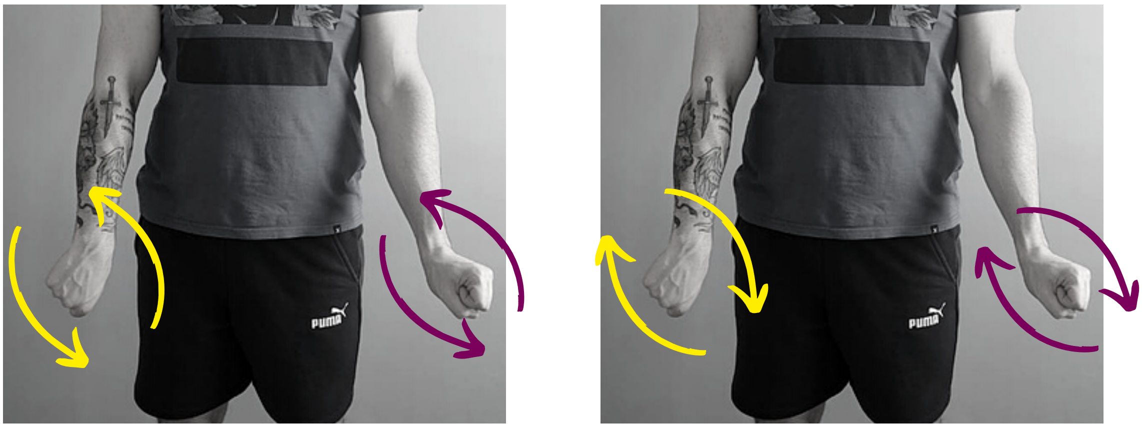

Directions: With both your arms and shoulders relaxed, rotate your wrists outward and then inward for 30 seconds. Perform controlled movements in both directions. **Perform 2 sets.**

HIP ROTATION

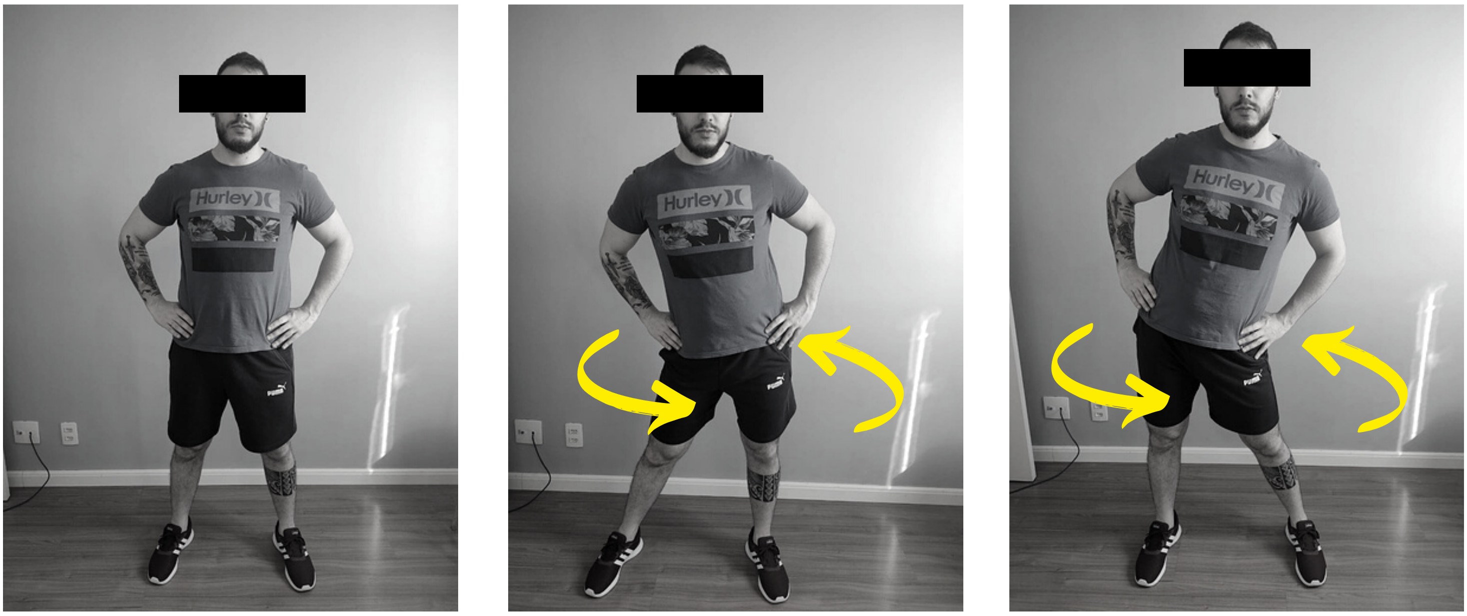

Directions: In a relaxed standing position, look straight ahead and place your hands on your hips. Perform controlled circular movements, rotating your hips 3 times to each side for 30 seconds. If necessary, place both hands on the wall to maintain balance. **Perform 2 sets.**

EXERCISE CARDS

PCFS grade: 4

STRENGTHENING EXERCISES

1. STANDING KNEE LIFTS

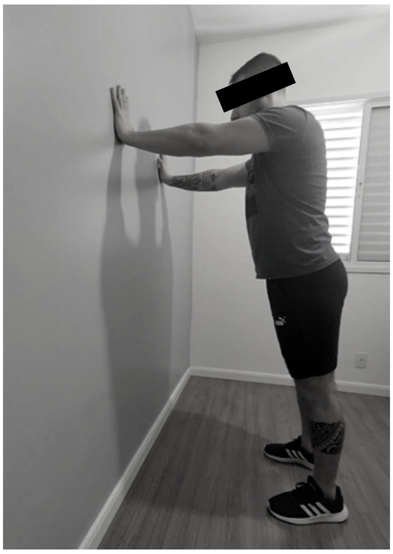

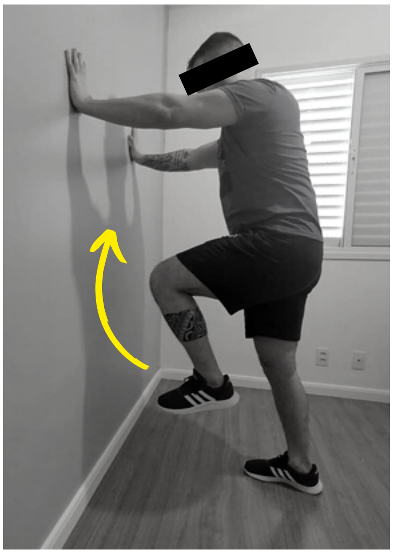

Directions: For greater balance and safety while performing the exercise, place both hands on the wall. In a controlled manner, raise your knee as high as you can and hold it for 2 seconds. Then return to the starting position and repeat the movement with the same leg. **Perform 3 sets of 10 to 15 repetitions for each leg.**

2. LYING SINGLE-LEG HIP FLEXION

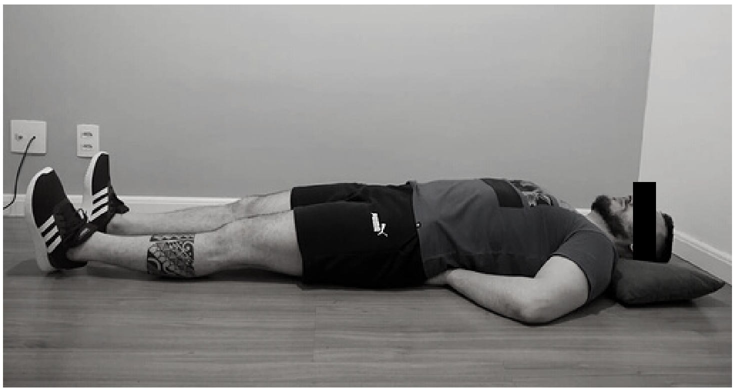

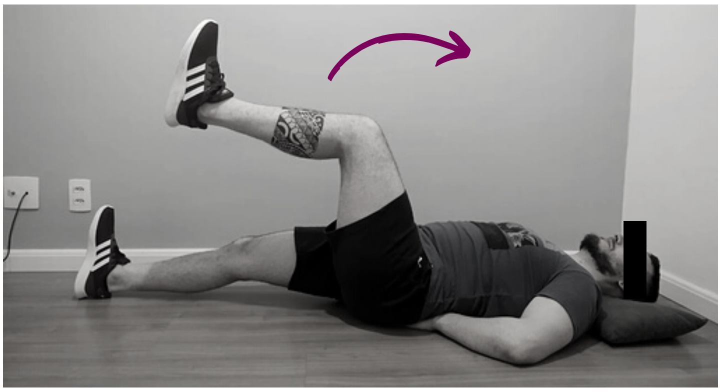

Directions: Lie down on a stable surface. Support your lumbar with both hands for comfort. Partially bend one knee and bring it toward your torso. Then return to the starting position and perform the movement with the other leg. Perform movements in a moderate range and in a controlled manner. **Perform 3 sets of 10 to 15 repetitions for each leg.**

EXERCISE CARDS

PCFS grade: 4

3. SEATED CALF-RAISES

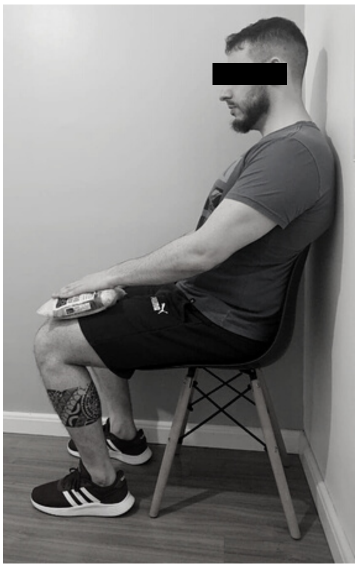

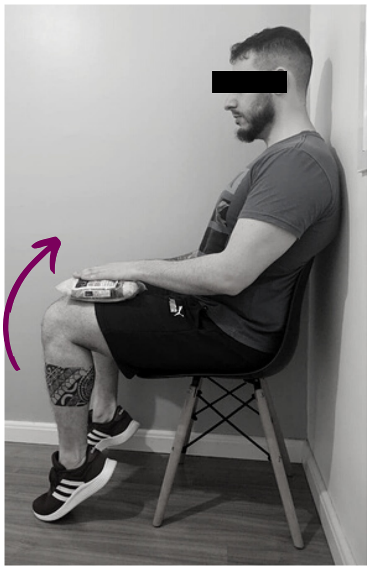

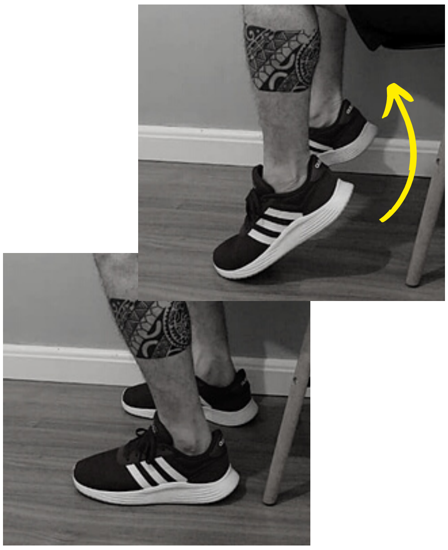

Directions: In a sitting position, place a weight (e.g., 2kg package) on your knees and lift both heels by pushing the floor with your toes. Hold the contraction for 2 seconds and return to the starting position. For safety, place the chair against a wall before starting the exercise. **Perform 3 sets of 10 to 15 repetitions.**

4. SEATED ELBOW FLEXION

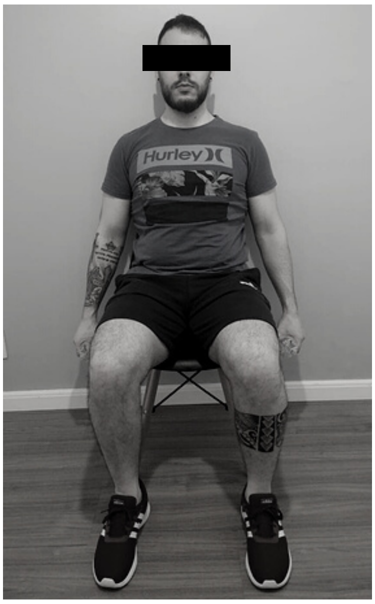

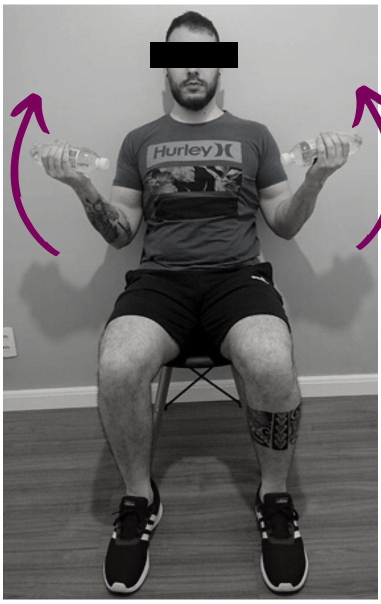

Directions: In a sitting position, hold a weight (e.g., 500ml bottle) in each hand and then flex your elbows to lift it. Hold the lifted weight for 2 seconds and return to the starting position. For safety, place the chair against a wall before starting the exercise. **Perform 3 sets of 10 to 15 repetitions.**

EXERCISE CARDS

PCFS grade: 4

5. LYING DIAPHRAGMATIC BREATHING

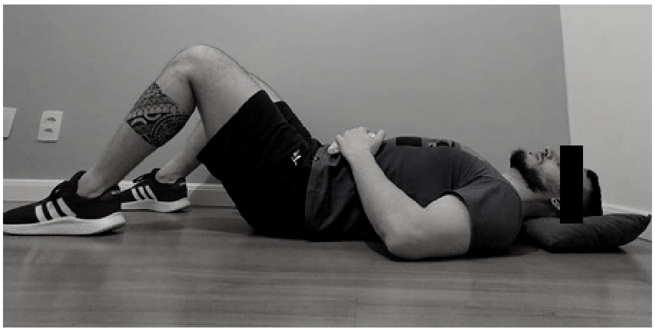

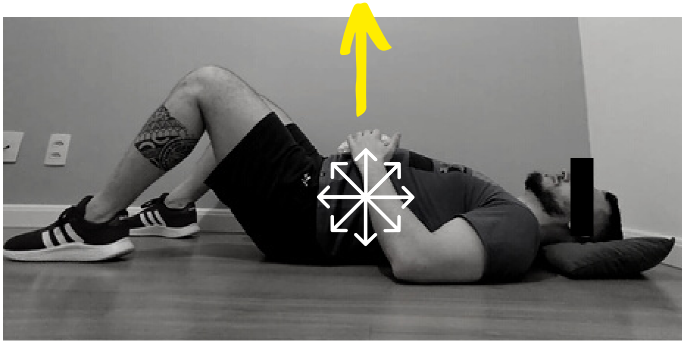

Directions: Lie down on a stable and comfortable surface. Place a weight (e.g., 1kg package) on your belly and rest both hands upon it. Inhale as much air as possible in a single breath, lifting the package and sustain it for 2 seconds. Then, exhale slowly and relax. **Perform 3 sets of 10 to 15 repetitions.**

6. SEATED ISOMETRIC HIP FLEXION

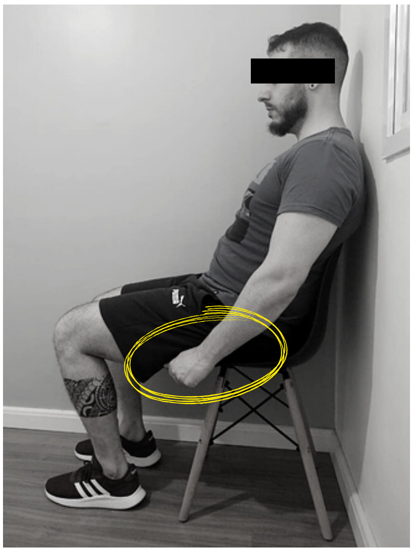

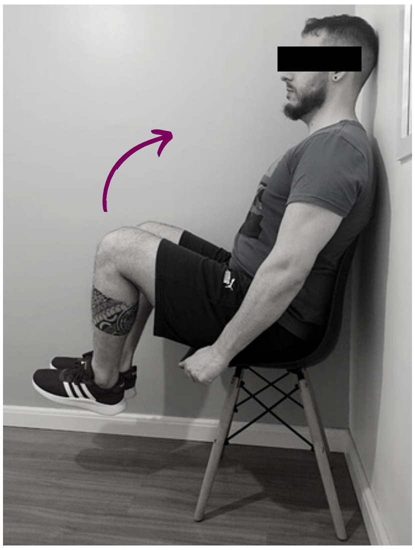

Directions: In a sitting and slightly reclined position, hold the front of the chair firmly for stability. Thereafter, try to lift both knees and feet (bringing them toward your torso). Hold them up for 15 to 30 seconds until you return to the starting position. If you can't sustain them raised for so long, do it for as long as you can. For safety, place the chair against a wall before start the exercise. **Perform 3 sets.**

EXERCISE CARDS

PCFS grade: 4

COOL-DOWN

NECK STRETCH

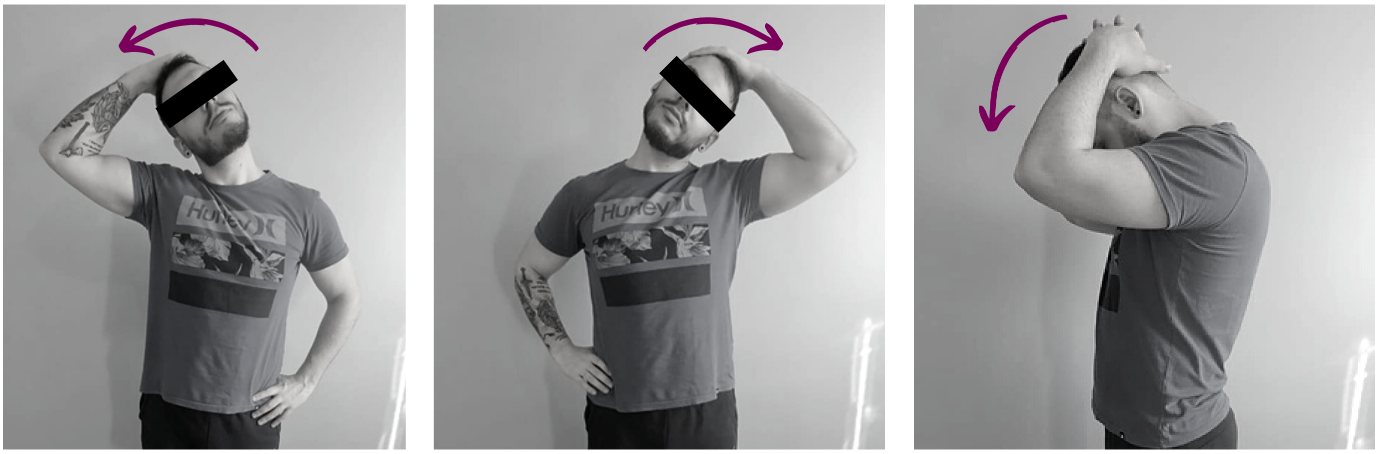

Directions: With your spine erect and stable, gently pull your head to one side and hold it for 20 to 30 seconds. Then, repeat it to the other side. Finally, with both hands, gently pull your head down. Exercise should be relaxing, so don't use excessive force!

ARMS STRETCH

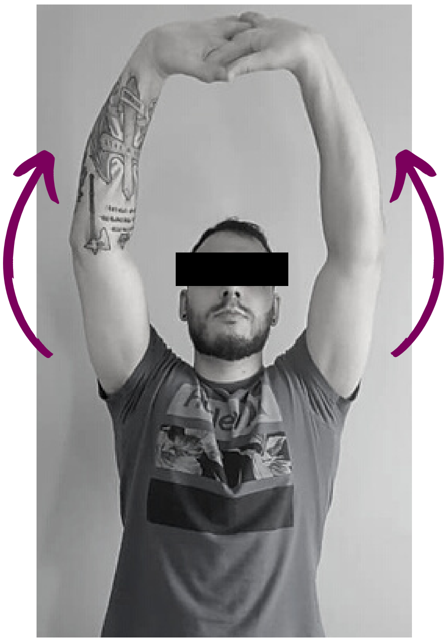

Directions: With your spine straight and stable, interlace your fingers and turn your palms outward. Raise your arms above your head (or as high as you can) and extend them for 20 to 30 seconds.

EXERCISE CARDS

PCFS grade: 3

WARM-UP

ARM SWINGS

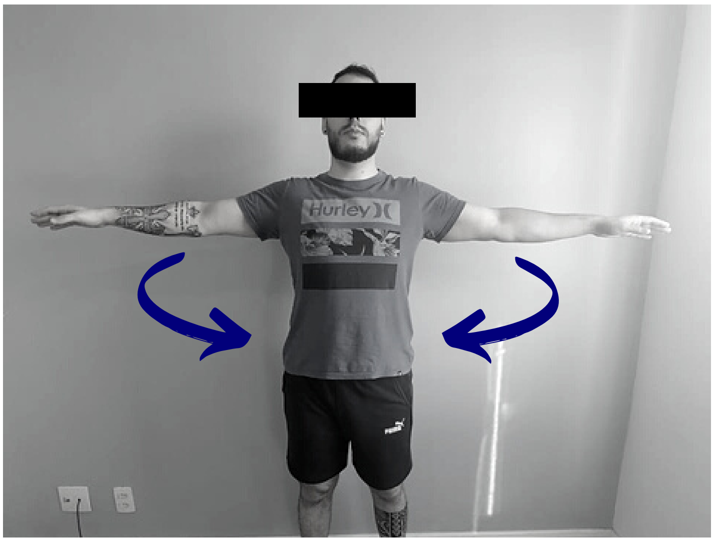

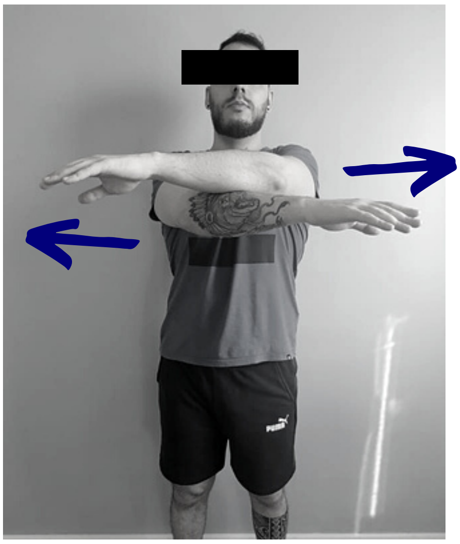

Directions: In a standing position, and with both arms relaxed, swing them parallel to the floor in a controlled way for 30 seconds. Your arms shall cross in front of your body and, then, return to the side of your body. **Perform 2 sets.**

HIP ROTATION

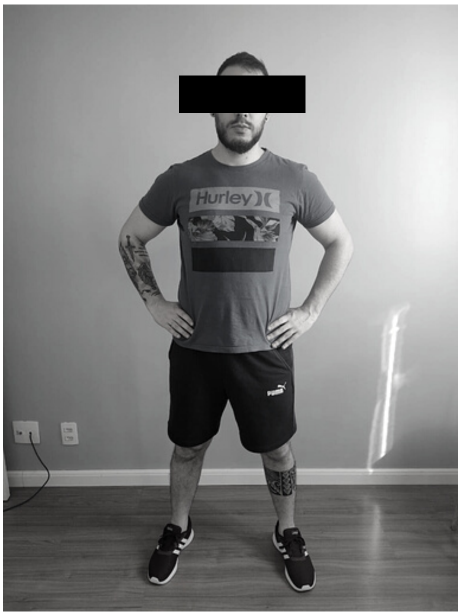

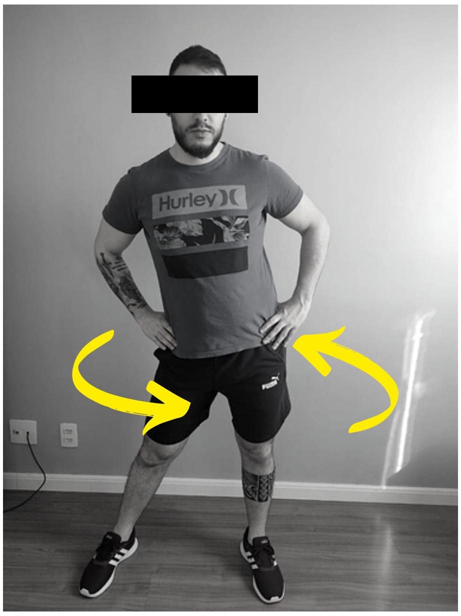

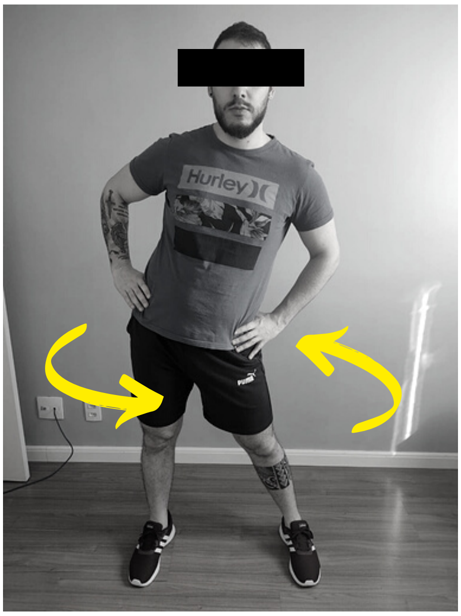

Directions: In a relaxed standing position, look straight ahead and place your hands on your hips. Perform controlled circular movements, rotating your hips 3 times to each side for 30 seconds. If necessary, place both hands on the wall to maintain balance. **Perform 2 sets.**

EXERCISE CARDS

PCFS grade: 3

STRENGTHENING EXERCISES

1. HIP RAISES

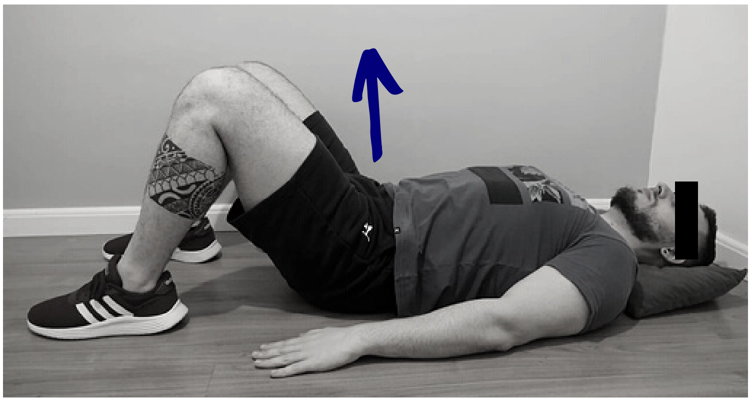
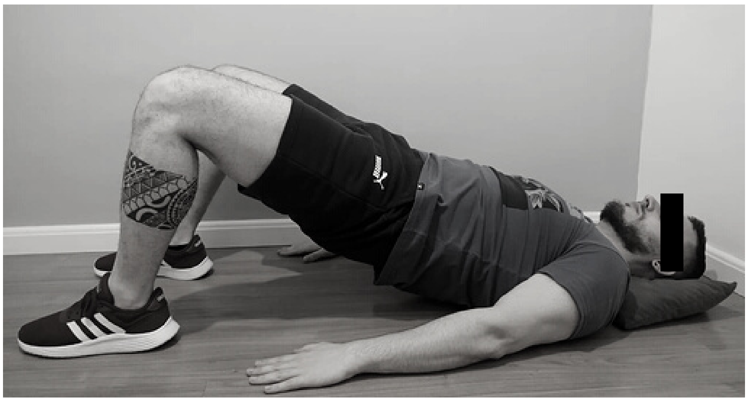

Directions: Lie down on a stable and comfortable surface. Bend your knees and place both feet firmly on the floor. For greater stability, rest both hands at your sides. In a controlled manner, lift your hip by contracting your glutes and legs' muscles. Hold your hip raised for 2 seconds and, then, return to the starting position. **Perform 3 sets of 10 to 15 repetitions.**

2. KNEE EXTENSION

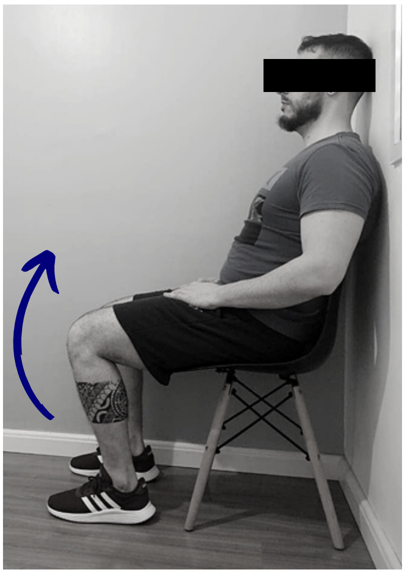
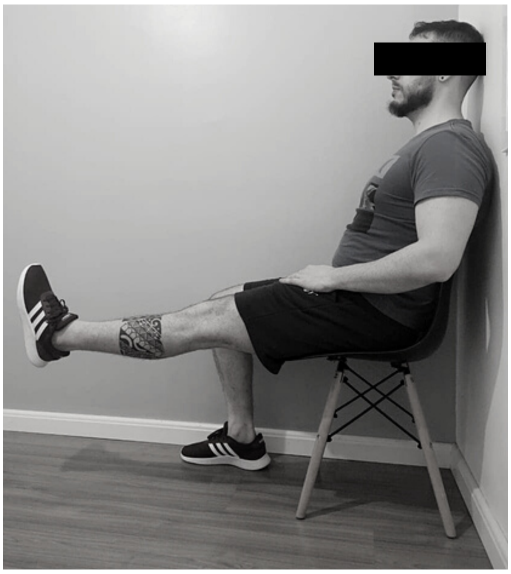

Directions: In a sitting position, contract the muscles of your leg to extend your knee. Hold your foot raised for a few seconds and return to the starting position. If it is too light, you can attach a weight (e.g., grocery bag + packages) to your ankle (when in doubt, please consult your trainer). For safety, place the chair against a wall before starting the exercise. **Perform 3 sets of 10 to 15 repetitions for each leg.**

EXERCISE CARDS

PCFS grade: 3

3. HIP ABDUCTION

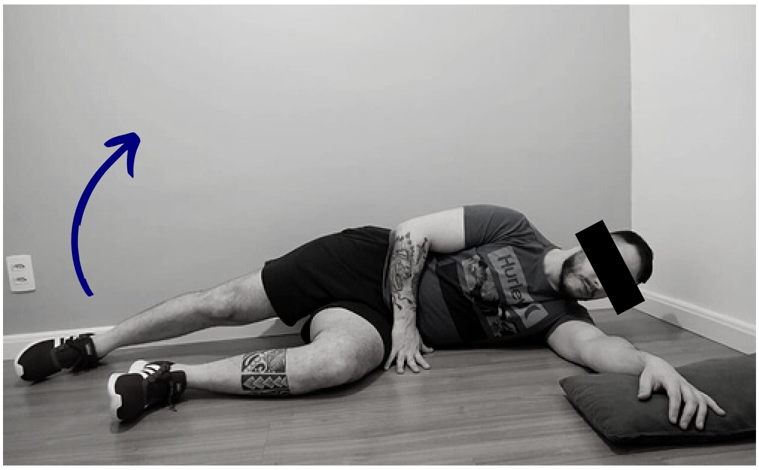

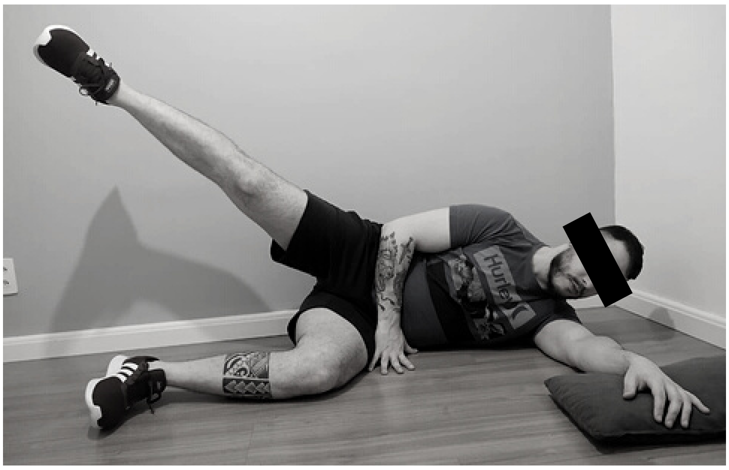

Directions: Lie on your side on a stable and comfortable surface. Bend your knee that is closer to the floow and extended the corresponding arm in front of your body for greater stability. Then, raise the other leg, moving it sideways. Hold your leg up for 2 seconds and return to the starting position. **Perform 3 sets of 10 to 15 repetitions for each leg.**

4. LATERAL RAISES

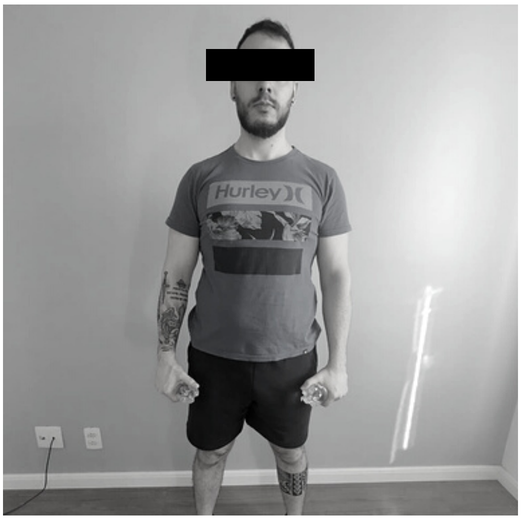

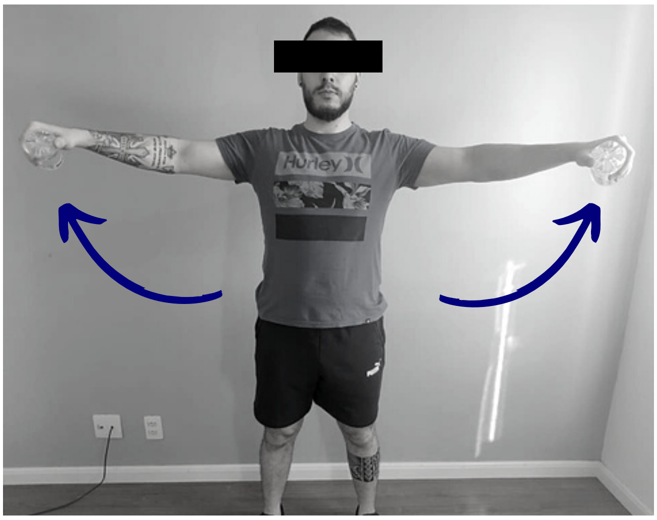

Directions: In a standing position, hold a weight (e.g., 500ml bottle) in each hand. Then raise both arms at your sides, bringing them up to shoulder height. Hold the weight lifted for 2 seconds and return to the starting position. **Perform 3 sets of 10 to 15 repetitions.**

EXERCISE CARDS

PCFS grade: 3

5. LYING TRICEPS EXTENSION

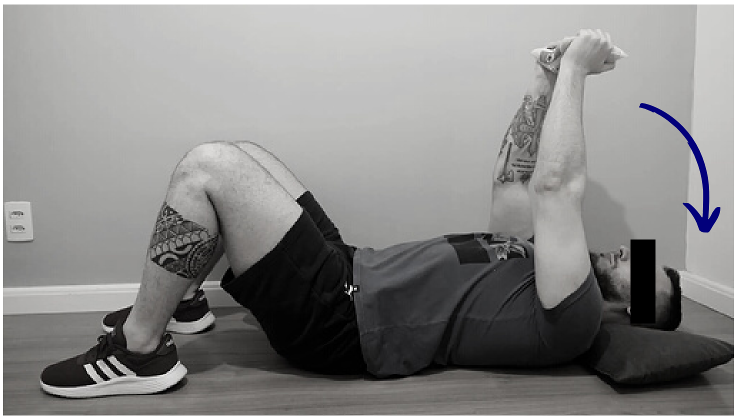

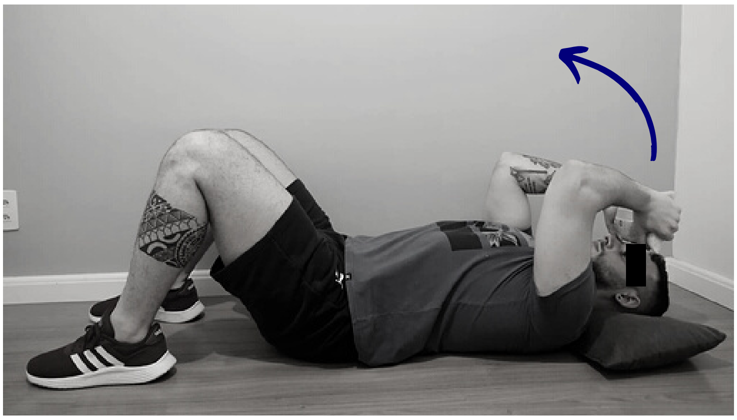

Directions: Lie down on a stable and comfortable surface. Initially, with both arms extended towards the sky (i.e., perpendicular to the ground), hold firmly a weight (e.g., 5kg package) with both hands. In a controlled manner, bend both elbows to bring the weight towards your forehead. Then, extend your elbows once again, returning to the starting position. **Perform 3 sets of 10 to 15 moves.**

6. CRUNCHES

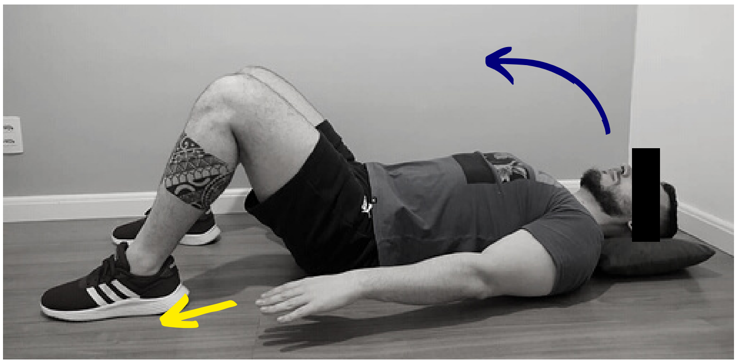

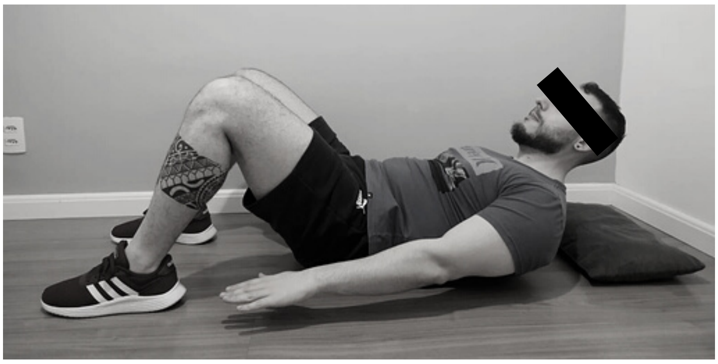

Directions: Lie down on a stable and comfortable surface. Bend your knees and place your feet firmly on the floor. With your arms extended by your sides, contract your abdominal muscles to lift your torso while bringing your hands toward your heels. Try to hold your torso raised for 2 to 3 seconds, then return to the starting position. **Perform 3 sets of 10 to 15 reps.**

EXERCISE CARDS

PCFS grade: 3

COOL-DOWN

GLUTE STRETCH

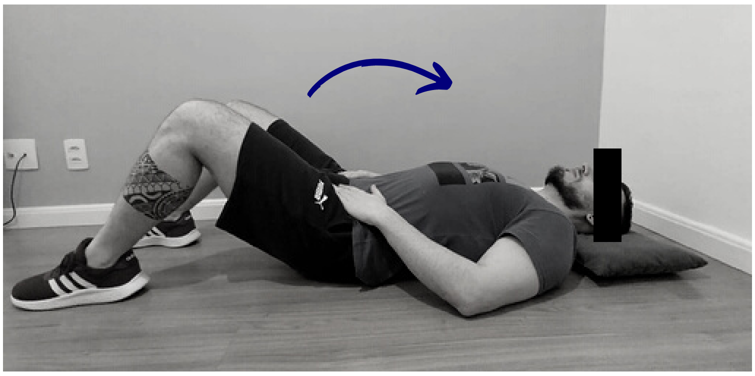
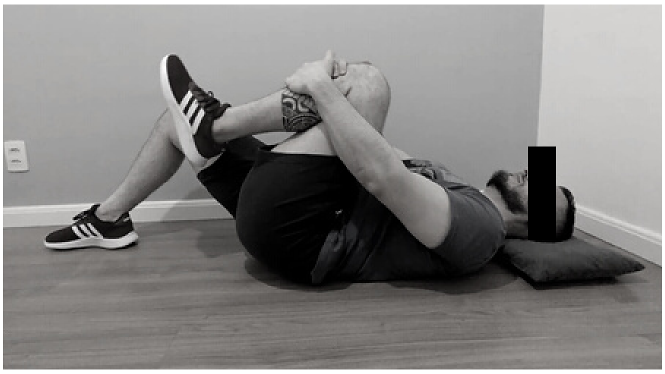

Directions: Lie down on a stable and comfortable surface. Bend your knees and place your feet firmly on the floor. Grab one of your knees and bring it towards your body. Hold it in a stretch position for 20 to 30 seconds. Then, return to the starting position and perform the movement with the opposite leg. This exercise should be relaxing, so don't exert excessive force!

SIT AND REACH

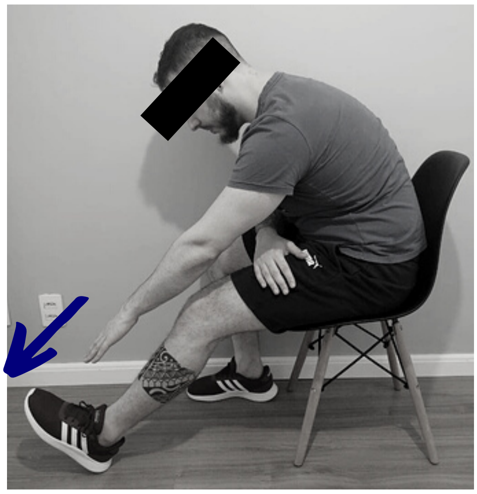
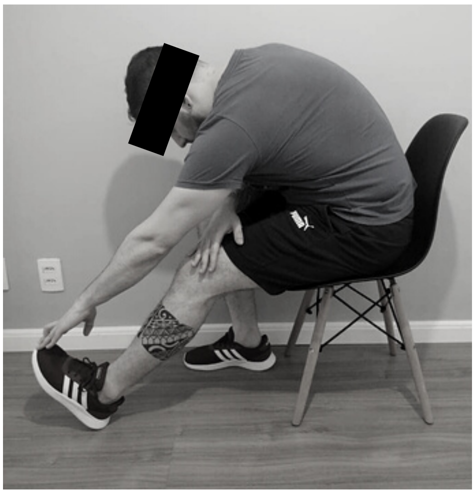

Instructions: In a sitting position, extend one of your knees and, in a slow movement, try to reach your toes with your hand from the same side. Stretch as much as you can and hold this position for 20 to 30 seconds. Then, return to the starting position and perform the movement with the opposite leg. For safety, place the chair against a wall or stable structure before start the exercise. This exercise should be relaxing, so don't exert excessive force!

EXERCISE CARDS

PCFS grade: 3

NECK STRETCH

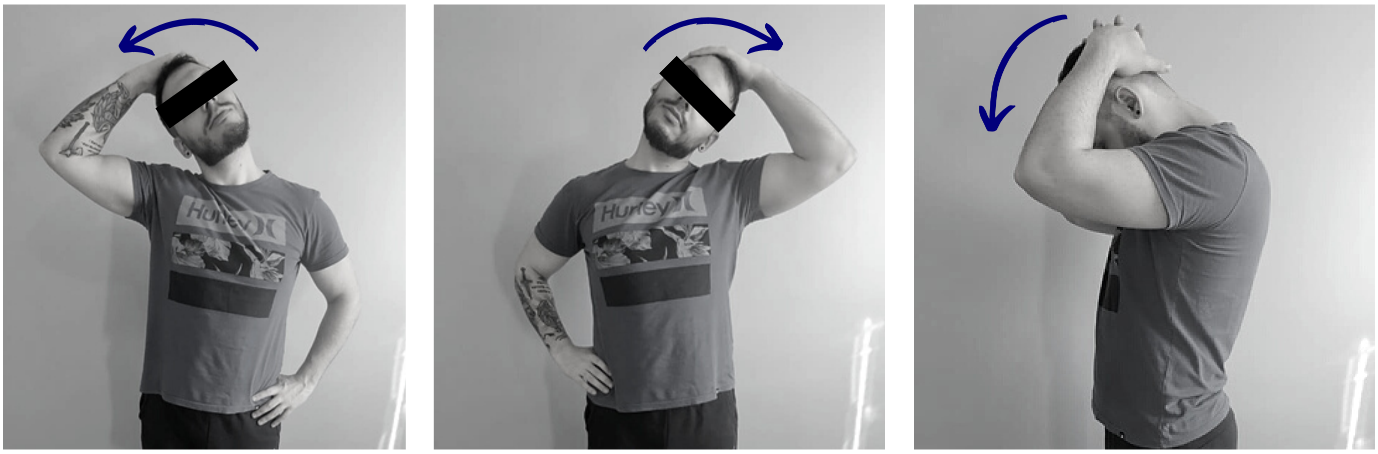

Directions: With your spine erect and stable, gently pull your head to one side and hold for 20 to 30 seconds. Then, repeat it to the other side. Finally, with both hands, gently pull your head down. Exercise should be relaxing, so don't use excessive force!

ARMS STRETCH

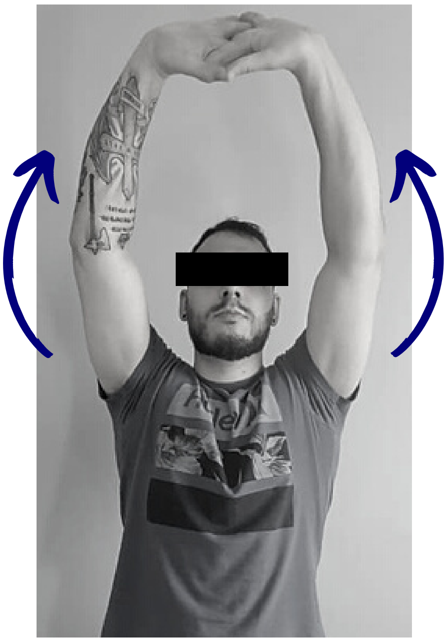

Directions: With your spine straight and stable, interlace your fingers and turn your palms outward. Raise your arms above your head (or as high as you can) and extend them for 20 to 30 seconds.

EXERCISE CARDS

PCFS grade: 2

WARM-UP

ARM SWINGS

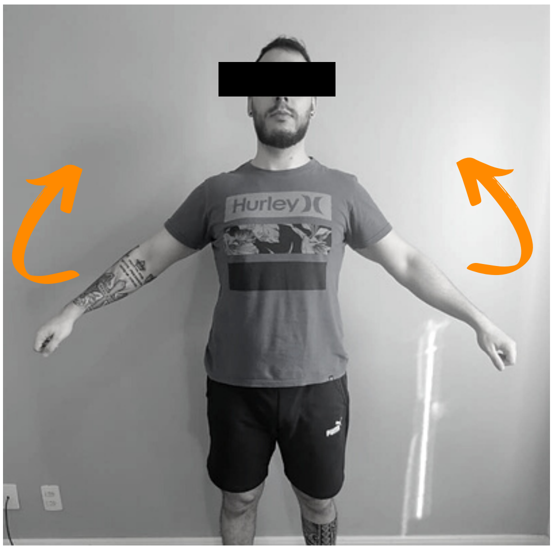

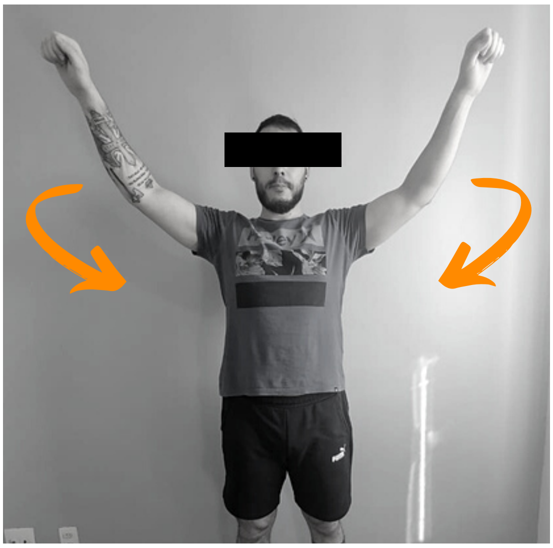

Directions: With arms relaxed and at your sides, rotate them back and forth in a controlled manner for 30 seconds. **Perform 2 sets.**

STANDING KNEE LIFT

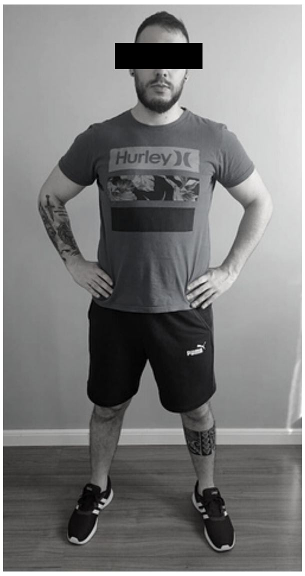

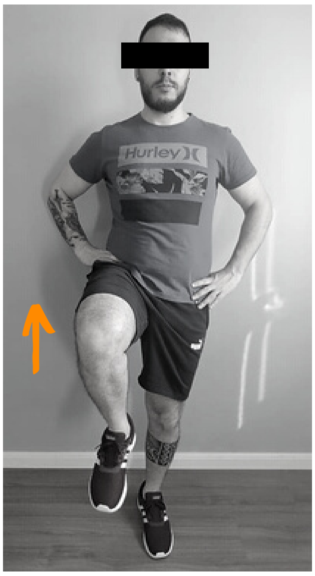

Directions: In a standing position, look straight ahead and place your hands on your hips. Raise your knees alternately as high as you can for 30 seconds. **Perform twice.**

EXERCISE CARDS

PCFS grade: 2

STRENGTHENING EXERCISES

1. SIT-TO-STAND

Directions: In a sitting position, place your feet close to the base of the chair. Lean your torso slightly forward and then lift your body in a single movement by using the strength of your leg muscles. Keep both hands resting on your shoulders. For safety, place the chair against a wall before starting the exercise.

Perform 4 sets of 10 to 15 repetitions.

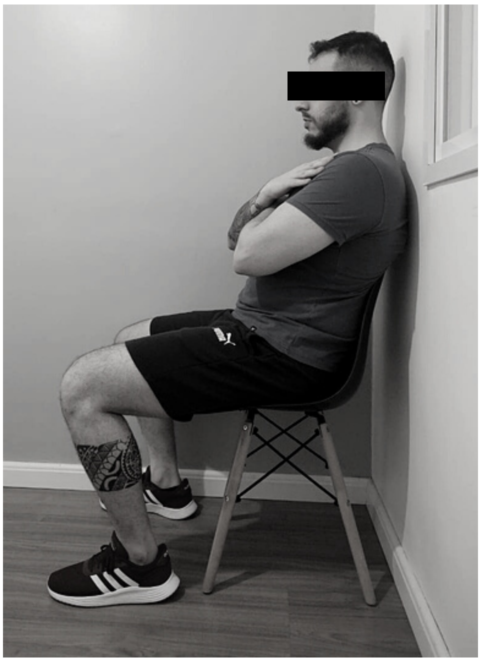

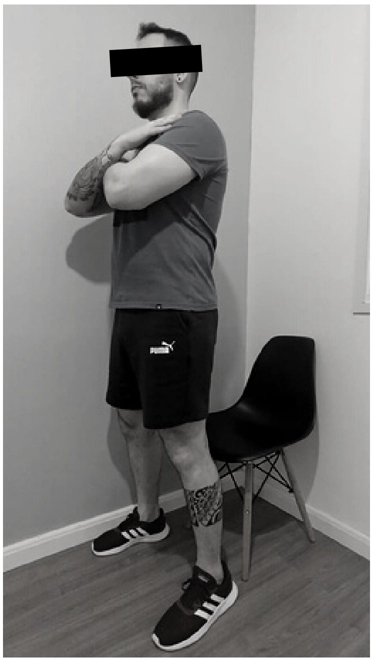

2. STEP-UP

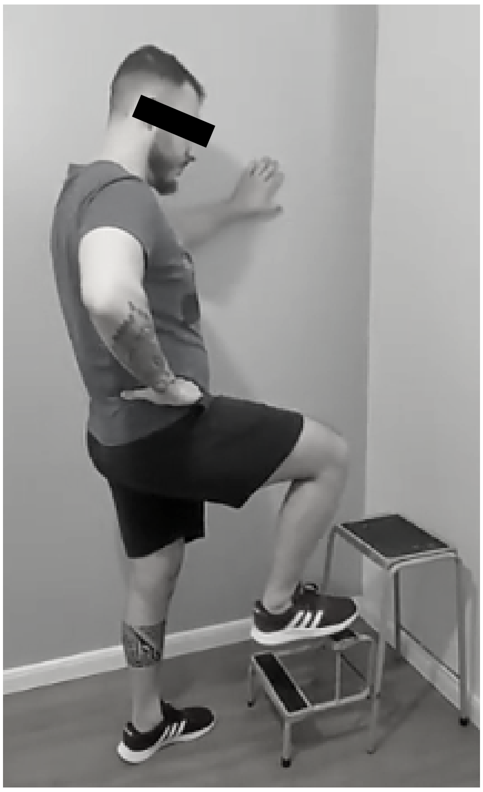

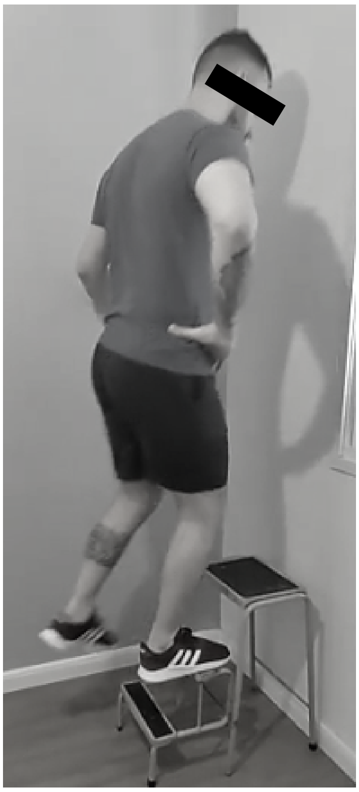

Directions: To perform this exercise, use a ladder or proper box (consult your trainer). Place a foot over the step while keeping the other leg on the floor. Raise your body as if you are climbing the stairs by applying force to the object through the strength of the leg muscles that are over it. Thereafter, return to the starting position. For safety, place one hand on the wall or handrail.

Perform 4 sets of 10 to 15 repetitions for each leg.

EXERCISE CARDS

PCFS grade: 2

3. FARMER WALK

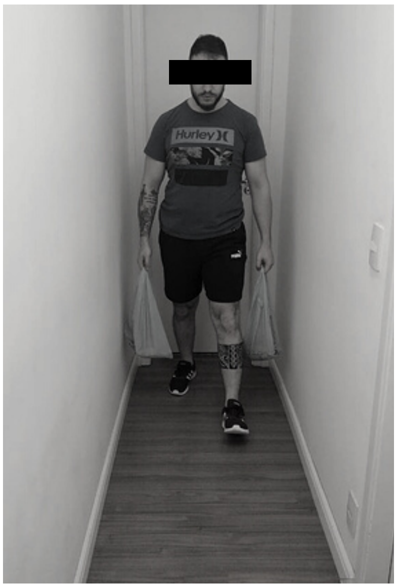

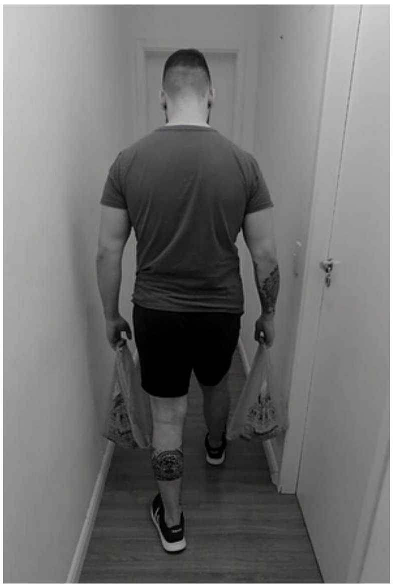

Directions: To perform this exercise, you will need two bags loaded with objects of same weight (e.g., 5kg packages). Walk for 45 to 60 seconds carrying both bags as shown in the images.

Perform 4 sets.

4. ELBOW FLEXION

Directions: Standing, hold a weight (e.g., bucket full of water) with a single hand. Then flex your elbow, bringing the lifted weight nearly to shoulder height. Hold the lifted weight for 2 seconds and return to the starting position. Rest your other hand on your hip for greater stability.

Perform 4 sets of 10 to 15 repetitions for each arm.

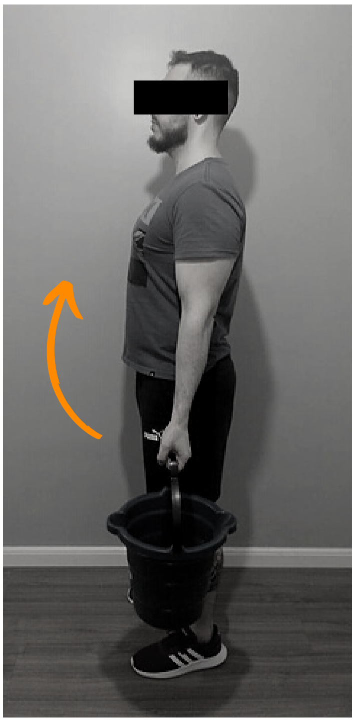
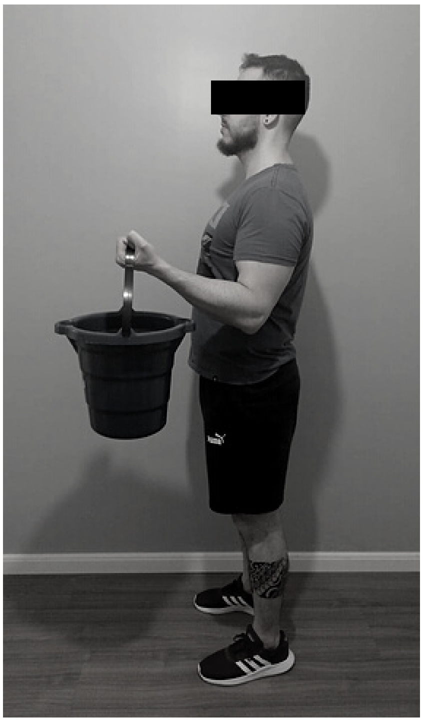

EXERCISE CARDS

PCFS grade: 2

5. OVERHEAD SHOULDER PRESS

Directions: In a standing position, hold a weight (e.g., 5kg package) with both hands at chest level (as shown in the picture). Then push the weight up and hold it raised for 2 seconds before returning to the starting position.

Perform 4 sets of 10 to 15 repetitions.

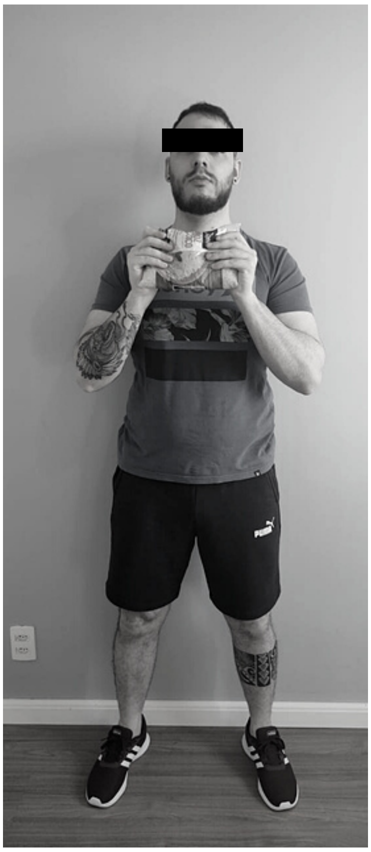

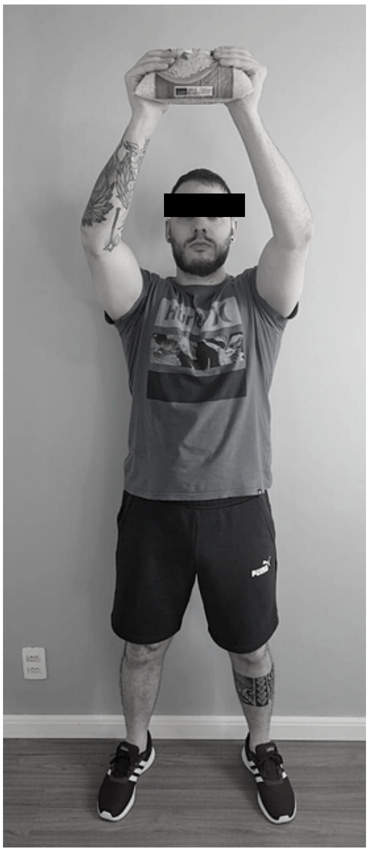

6. LYING HIP FLEXION

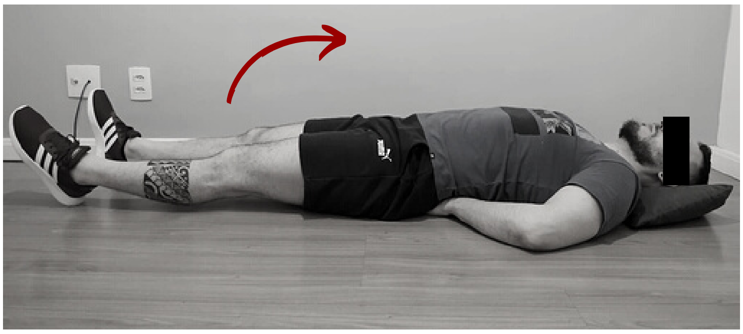

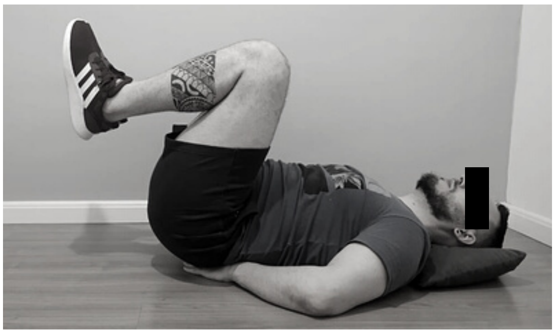

Directions: Lie down on a stable and comfortable surface. Support your lumbar with both hands for comfort. Then bend both knees and move them toward your torso by contracting your abdominal muscles.

Perform 4 sets of 10 to 15 repetitions.

EXERCISE CARDS

PCFS grade: 2

COOL-DOWN

SHOULDER STRETCH

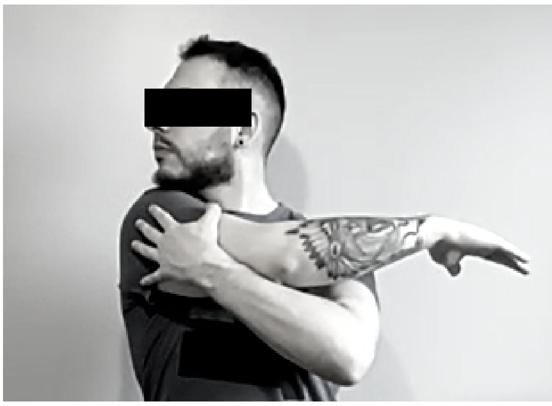

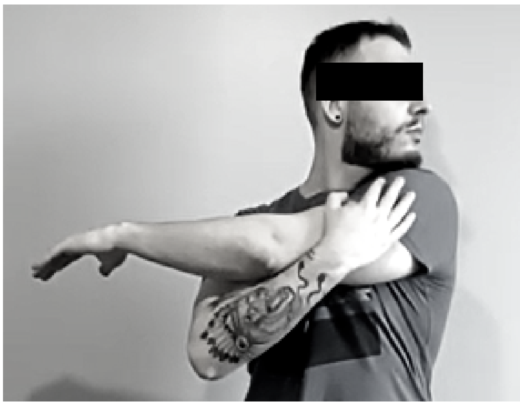

Directions: In a standing position, cross one arm in front of your body at shoulder height. Then pull your elbow with your other arm. Hold this position for 20 to 30 seconds. Then, return to the starting position to perform the same movement with the other side. During the movement, rotate your neck, turning your head to the opposite side. The exercise should be relaxing, so don't exert excessive force!

SIT AND REACH

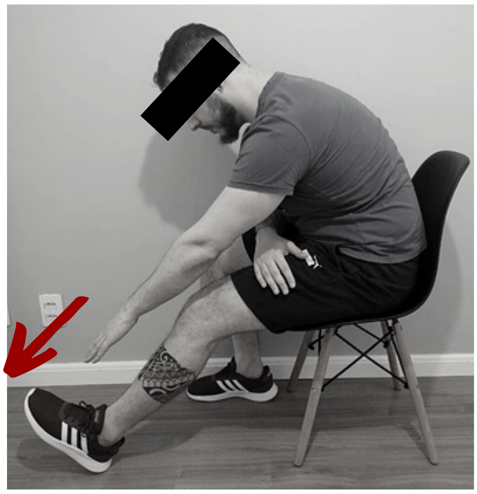

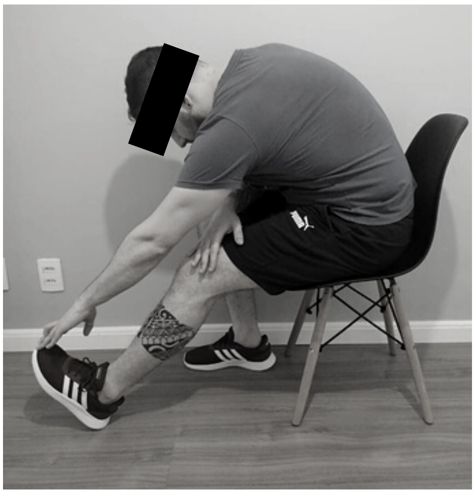

Instructions: In a sitting position, extend one of your knees and, in a slow movement, try to reach your toes with your hand from the same side. Stretch as much as you can and hold this position for 20 to 30 seconds. Then, return to the starting position and perform the movement with the opposite leg. For safety, place the chair against a wall or stable structure before start the exercise. This exercise should be relaxing, so don't exert excessive force!

EXERCISE CARDS

PCFS grade: 2

DORSAL STRETCH

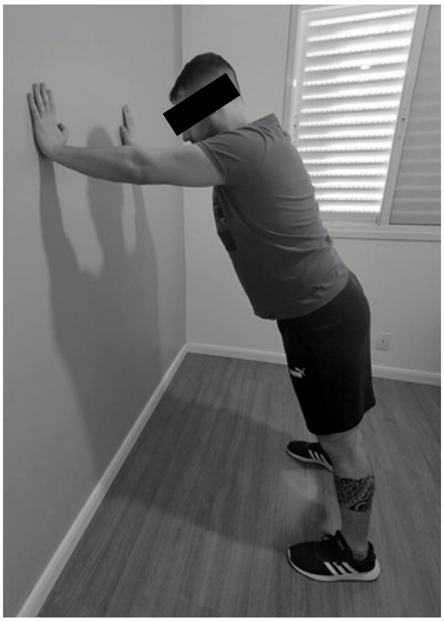
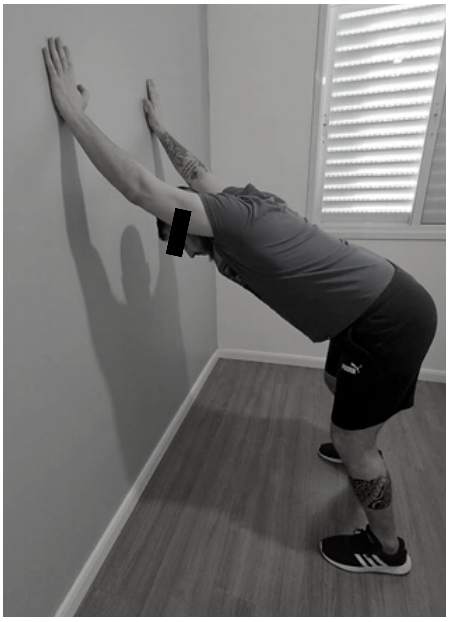

Directions: Standing at a distance of approximately 1m, place both hands on the wall at eye level. Then slowly move your hip backwards, bending your torso forward. Stretch your back muscles for 20 to 30 seconds and return to starting position. The exercise should be relaxing, so don't exert excessive force!

QUADRICEPS STRETCH

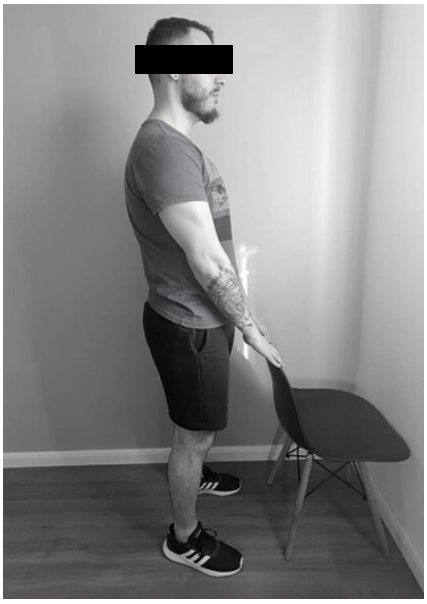
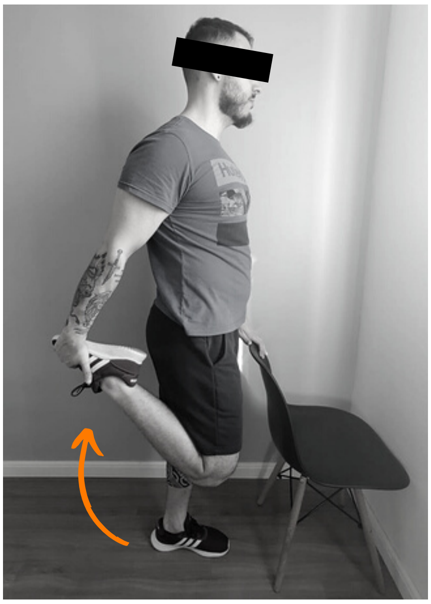

Directions: Standing next to a stable structure (e.g., wall or chair), flex one knee and pull your ankle toward your back. Hold it for 20 to 30 seconds. Then, return to the starting position and perform the movement with the opposite leg.

EXERCISE CARDS

PCFS grade: 0/1

WARM-UP

ARM SWINGS

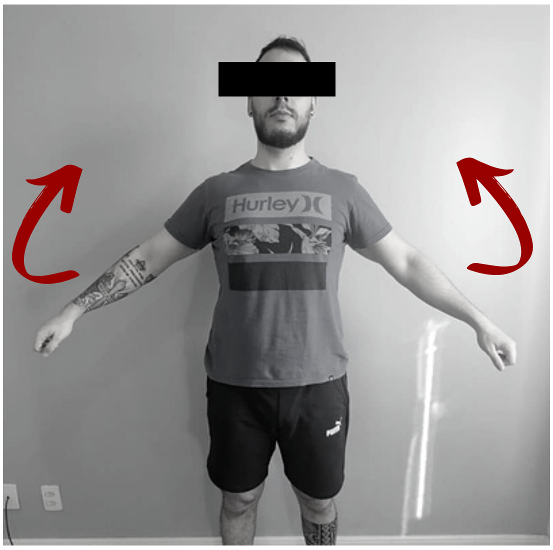

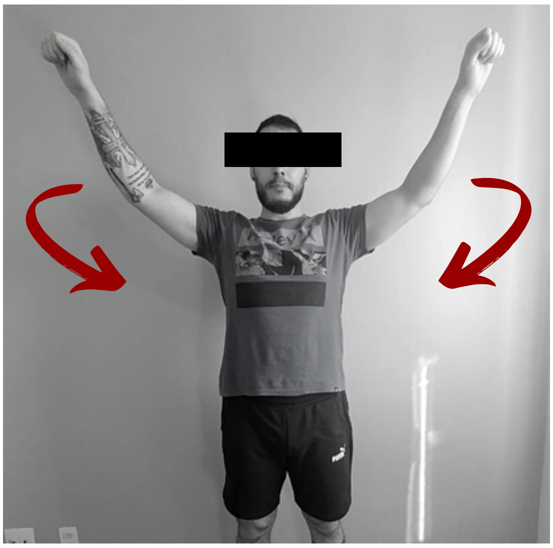

Directions: With arms relaxed and at your sides, rotate them back and forth in a controlled manner for 30 seconds. **Perform 2 sets.**

STANDING KNEE LIFT

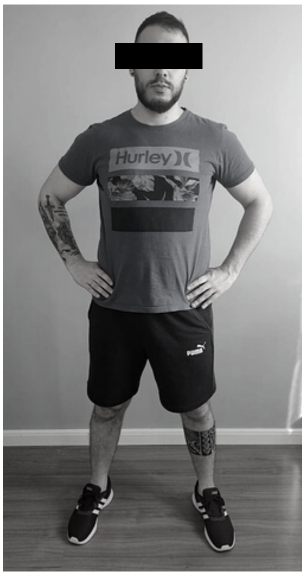

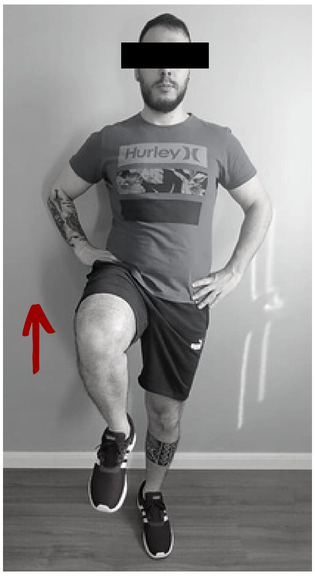

Directions: In a standing position, look straight ahead and place your hands on your hips. Raise your knees alternately as high as you can for 30 seconds. **Perform twice.**

EXERCISE CARDS

PCFS grade: 0/1

STRENGTHENING EXERCISES

1. HALF SQUAT

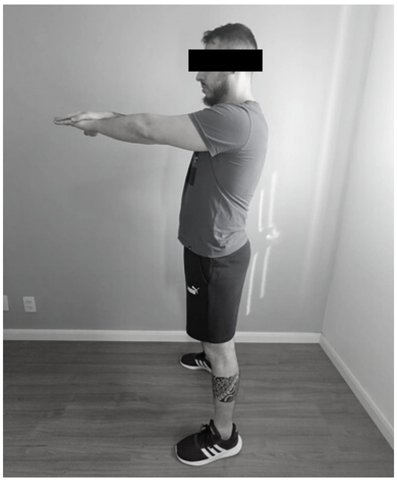

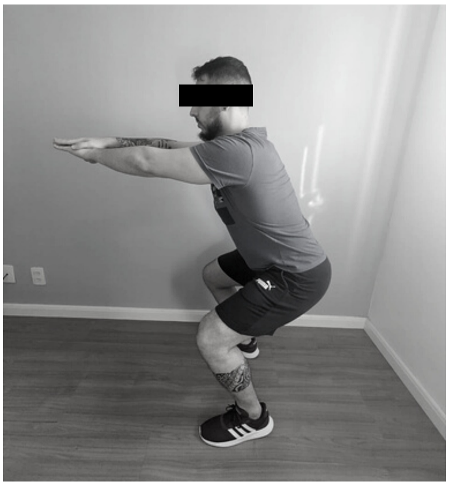

Directions: In a standing position, place your feet at shoulder width and extend your arms in front of your body, placing one hand over the other. Gently, bend your knees and evenly move your hips backwards. During this "downward" movement, try to keep your spine straight. Lower your body as much as you can, and then return to the starting position. **Perform 4 sets of 10 to 15 repetitions.**

2. LUNGES

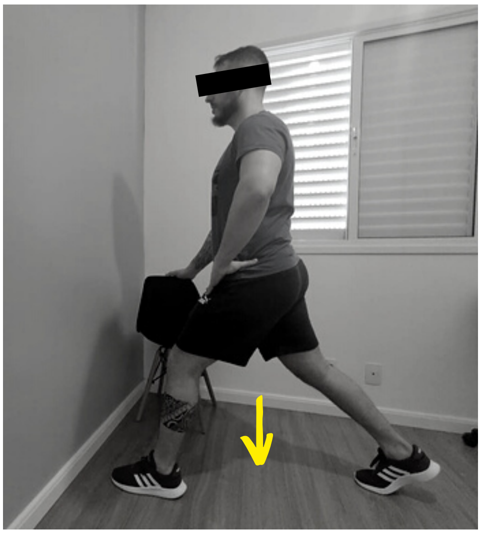

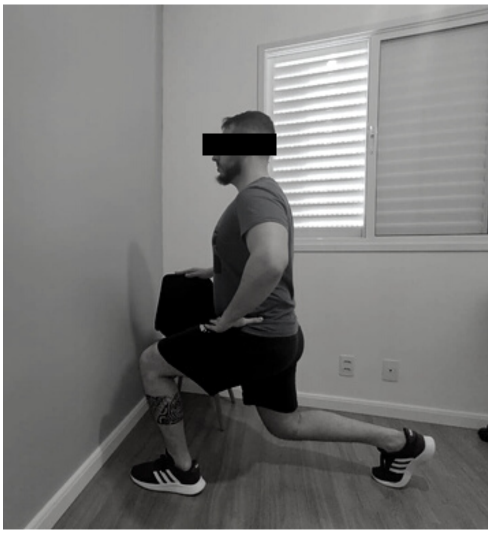

Directions: In a standing position, step forward with one of your legs. Bend the knee of the front leg and slowly lower your body until the knee of the hind leg almost touches the floor. Then, extend your knees to lift your body. During the exercise, try to focus the effort on your front leg. Use a chair (positioned against a wall) or a stable structure (e.g., handrail) for greater stability. **Perform 4 sets of 10 to 15 repetitions for each leg.**

EXERCISE CARDS

PCFS grade: 0/1

3. CALF-RAISES

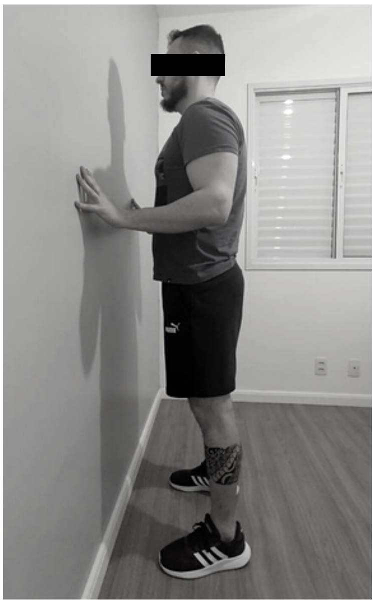
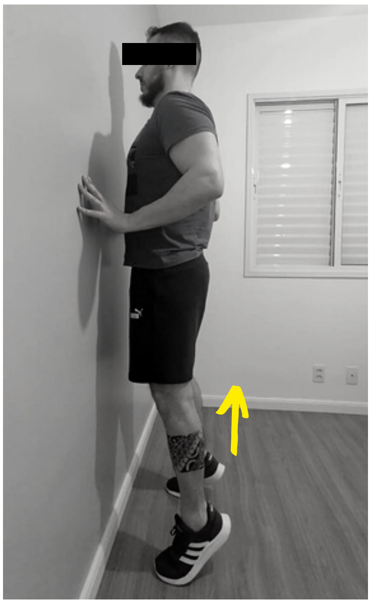

Directions: In a standing position, close to a wall, place both hands lightly upon it for greater stability during the exercise. In a single movement, lift your body weight by contracting calves to raise your heels. Try to push the floor with both feet. Then return to the starting position in a controlled manner. **Perform 4 sets of 10 to 15 movements.**

4. WALL PUSH-UP

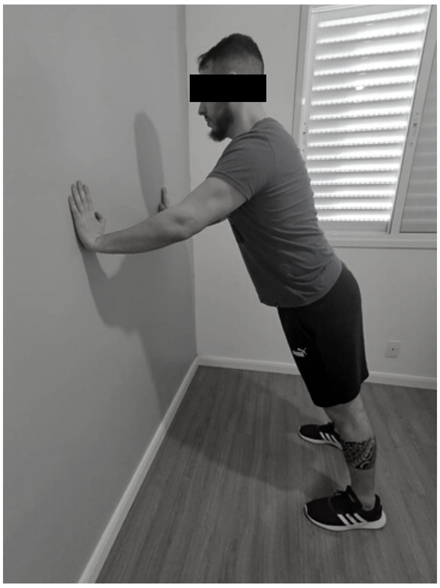
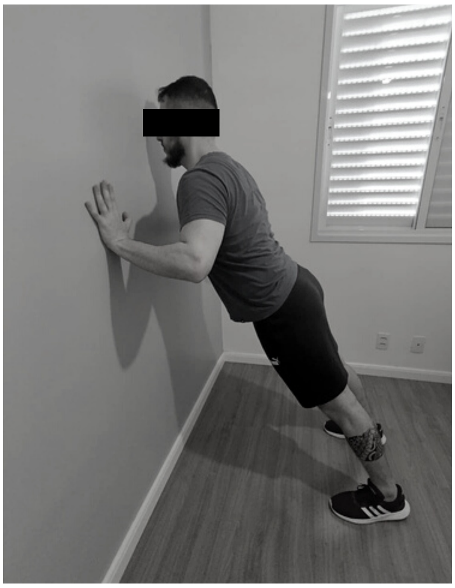

Directions: Stand approximately 1 meter away from a wall. Place both hands upon the wall, below the shoulder line, slightly leaning your body forward. In a controlled manner, flex both elbows. Then, after getting closer to the wall, extend your elbows by pushing the wall to return to the starting position. **Perform 4 sets of 10 to 15 movements.**

EXERCISE CARDS

PCFS grade: 0/1

5. STANDING ONE-ARM ROW

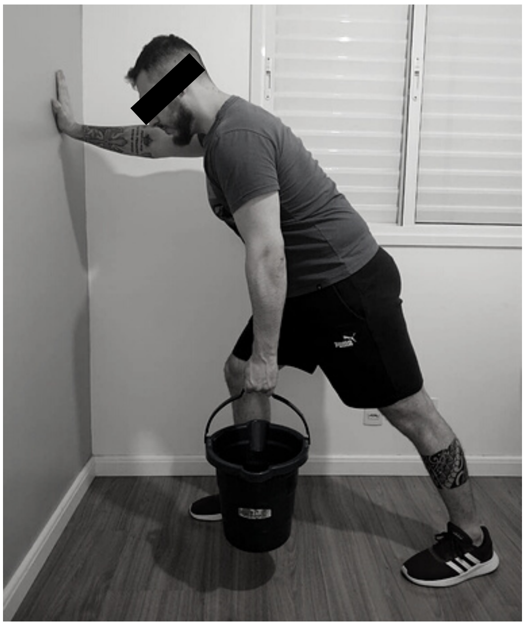

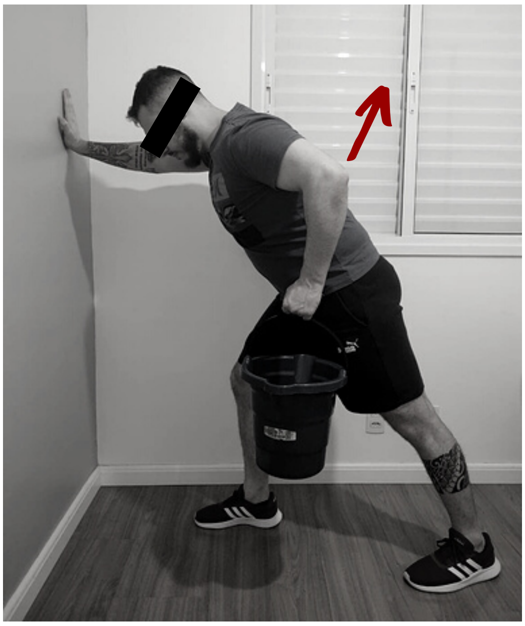

Directions: Stand approximately 1 meter away from a wall and step back with one of your legs. Place the hand of the same side as your front leg on the wall, at shoulder height, leaning yor torso slightly. With the opposite arm (same side as the back leg) hold a weight (e.g., bucket full of water) perpendicular to the ground. Pull the weight, bringing your elbow toward your waist. Then hold your elbow "elevated" for 2 seconds and return it towards the ground. **Perform 4 sets of 10 to 15 movements.**

6. PLANK

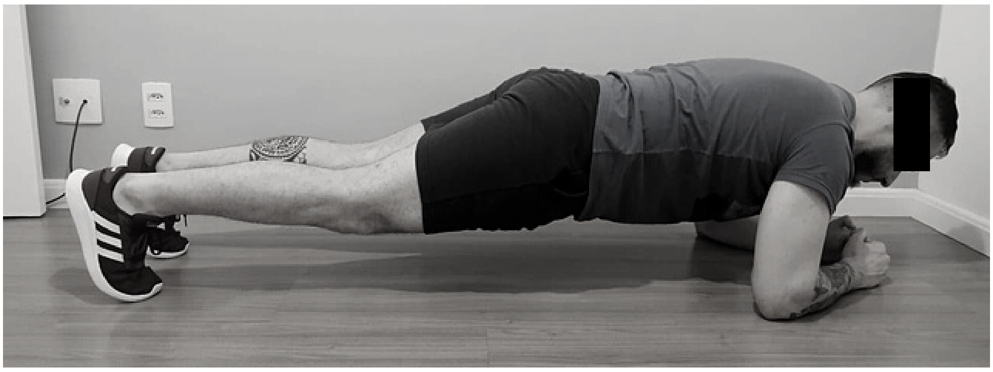

Directions: Lie face down and place your forearms on the floor, with your hands close together and your elbows apart, below the shoulder line. Lift your hip and keep your body aligned, keeping your weight distributed between your toes and forearms. Try to maintain the position by contracting the abdomen. **Perform 4 sets of 30 seconds.**

EXERCISE CARDS

PCFS grade: 0/1

COOL-DOWN

SHOULDER STRETCH

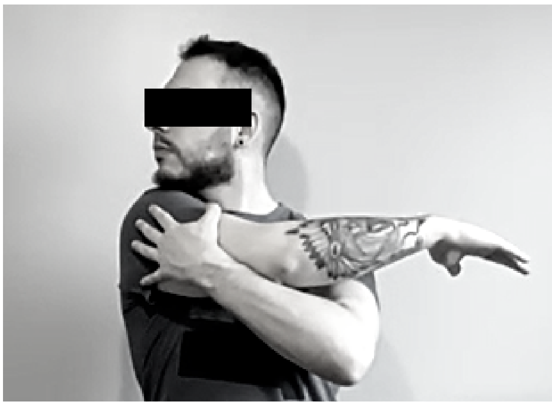
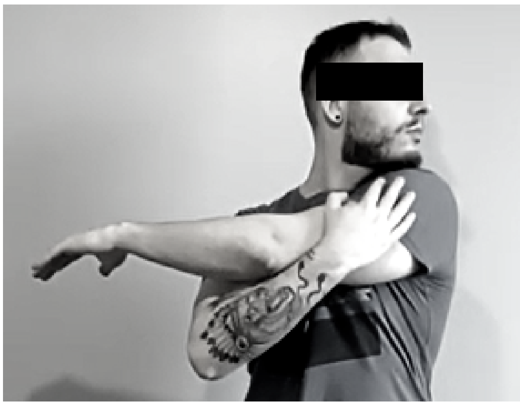

Directions: In a standing position, cross one arm in front of your body at shoulder height. Then pull your elbow with your other arm. Hold this position for 20 to 30 seconds. Then, return to the starting position to perform the same movement with the other side. During the movement, rotate your neck, turning your head to the opposite side. The exercise should be relaxing, so don't exert excessive force!

SIT AND REACH

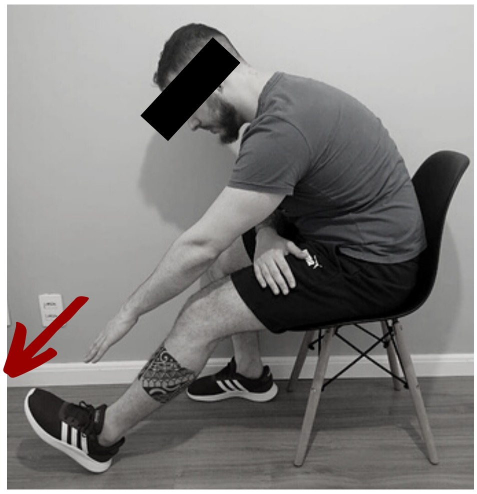
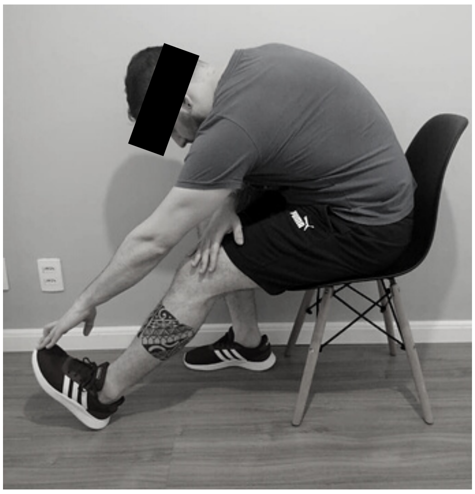

Instructions: In a sitting position, extend one of your knees and, in a slow movement, try to reach your toes with your hand from the same side. Stretch as much as you can and hold this position for 20 to 30 seconds. Then, return to the starting position and perform the movement with the opposite leg. For safety, place the chair against a wall or stable structure before start the exercise. This exercise should be relaxing, so don't exert excessive force!

EXERCISE CARDS

PCFS grade: 0/1

DORSAL STRETCH

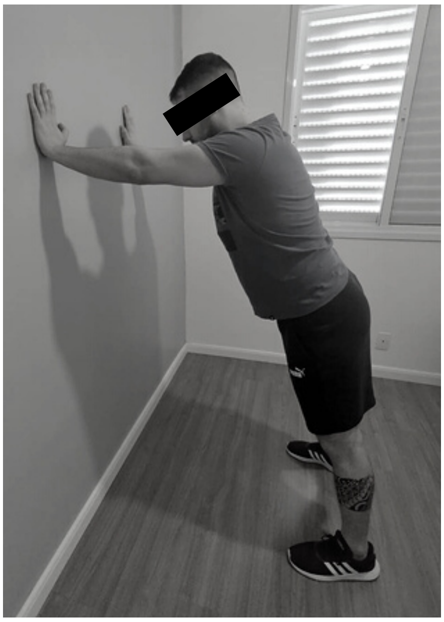
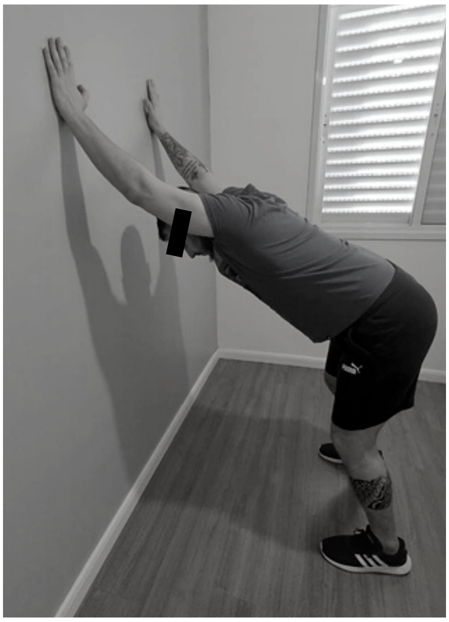

Directions: Standing at a distance of approximately 1m, place both hands on the wall at eye level. Then slowly move your hip backwards, bending your torso forward. Stretch your back muscles for 20 to 30 seconds and return to starting position. The exercise should be relaxing, so don't exert excessive force!

QUADRICEPS STRETCH

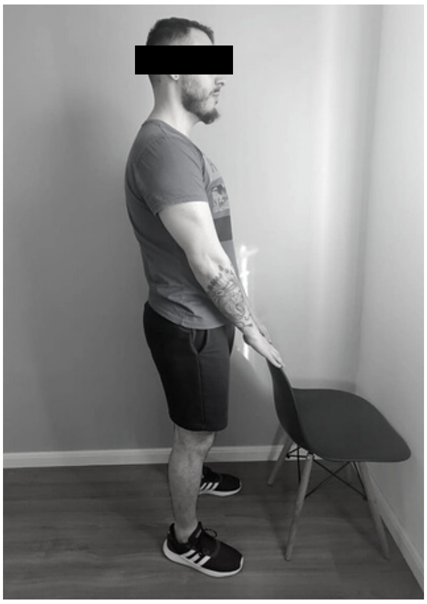
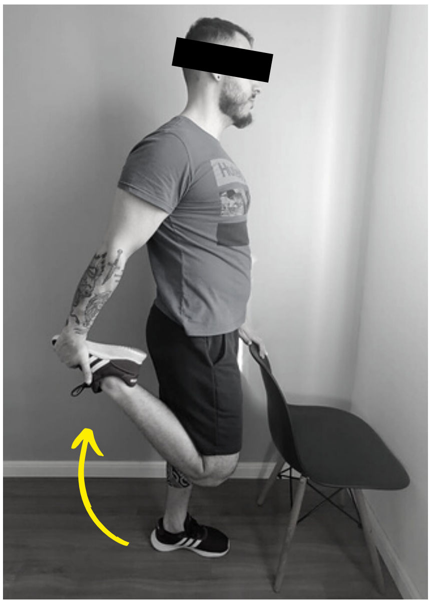

Directions: Standing next to a stable structure (e.g., wall or chair), flex one knee and pull your ankle toward your back. Hold it for 20 to 30 seconds. Then, return to the starting position and perform the movement with the opposite leg.
