# Supplementary material for: Benefits of Home-Based Exercise Training Following Critical SARS-CoV-2 Infection: A Case Report
Source: Front Sports Act Living. 2022 Jan 11;3:791703. doi: 10.3389/fspor.2021.791703 (PMC8787158; doi:10.3389/fspor.2021.791703)
Supplement: Supplementary Material 4 — is available at https://figshare.com/s/30f348a90e8d4faad445. [file Data_Sheet_4.PDF]

Supplementary Material 4

Supplementary Table 2. Home-based exercise training intensity progression.

Weeks	1 and 2	3 and 4	5 and 6	7 and 8	9 and 10
Target RPE	9-11	11-13	13-14	14-15	14-16

RPE: rate of perceived exertion
