# Supplementary material for: Benefits of Home-Based Exercise Training Following Critical SARS-CoV-2 Infection: A Case Report
Source: Front Sports Act Living. 2022 Jan 11;3:791703. doi: 10.3389/fspor.2021.791703 (PMC8787158; doi:10.3389/fspor.2021.791703)
Supplement: Supplementary Material 5 — is available at https://figshare.com/s/6d3dcda8da82a12c43c2. [file Data_Sheet_5.PDF]

Supplementary Material 5

Supplementary Table 3. Strengthening exercises according to each PCFS grade.

PCFS Grade	Exercises
4	1) Standing knee lifts<br>2) Lying single-leg hip flexion<br>3) Seated calf-raises<br>4) Seated elbow flexion<br>5) Lying diaphragmatic breathing<br>6) Seated isometric hip flexion
3	1) Hip raises<br>2) Knee extension<br>3) Hip abduction<br>4) Lateral raises<br>5) Lying triceps extension<br>6) Crunches
2	1) Sit-to-Stand<br>2) Step-up<br>3) Farmer Walk<br>4) Elbow flexion<br>5) Overhead shoulder press<br>6) Lying hip flexion
1 and 0	1) Half squat<br>2) Lunges<br>3) Calf-raises<br>4) Wall push-up<br>5) Standing one-arm row<br>6) Plank

PCFS: post-covid functional status
