# Supplementary material for: Benefits of Home-Based Exercise Training Following Critical SARS-CoV-2 Infection: A Case Report
Source: Front Sports Act Living. 2022 Jan 11;3:791703. doi: 10.3389/fspor.2021.791703 (PMC8787158; doi:10.3389/fspor.2021.791703)
Supplement: Supplementary Material 6 — is available at https://figshare.com/s/d91371b8c3fe90c7b732. [file Data_Sheet_6.PDF]

Supplementary Material 6

1. Procedures

1.1 Post-COVID Functional Status Scale

The Brazilian Portuguese version of the Post-COVID Functional Status (PCFS) scale access functional impairment during follow-up after COVID-19 infection. The instrument consists in 17 binary (yes or no) questions, each with a specific score varying between 0 and 4. Grade 0 reflects the absence of any functional limitation; Grade 1 indicates that symptoms, pain or anxiety are present to an increasing degree, but there is no impediment or limitation in performing any daily activity; Grade 2 indicates that the patient is capable of performing daily activities (including social roles) but at a lower intensity; Grade 3 indicates that the patient presents inability to perform certain activities, forcing them to structurally modify these and; Grade 4 is reserved for those patients with severe functional limitations, requiring assistance with activities of daily living. Overall classification is based on the highest-scoring answer, and reflects the functional status of the patient. For the application of the questionnaire, a structured interview was performed, following the authors' recommendations.[1]

1.2 Fatigue Severity Scale

The Fatigue Severity Scale (FSS) was developed with the purpose of evaluating the functional performance related to fatigue. We used the translated (Brazilian Portuguese) and validated version of the FSS.[2] This is a 9-item multidimensional scale that covers the physical, social or cognitive effects of fatigue (e.g., function, work, motivation). For each item, the answer ranges from 1 (Strongly Disagree) to 7 (Strongly Agree), according to a Likert scale. Higher scores reflect greater fatigue. All items were scored, summed and then averaged for an overall score.[3]

2. References

1. Klok FA, Boon G, Barco S, Endres M, Geelhoed JJM, Knauss S, et al. The Post-COVID-19 Functional Status scale: a tool to measure functional status over time after COVID-19. Eur Respir J. 2020;56(1).

2. Toledo FO, Junior WM, Speciali JG, Sobreira C. PND66 Cross-cultural adaptation and validation of the Brazilian version of the Fatigue Severity Scale (FSS). Value in Health. 2011;7:2.

3. Krupp LB, LaRocca NG, Muir-Nash J, Steinberg AD. The fatigue severity scale. Application to patients with multiple sclerosis and systemic lupus erythematosus. Arch Neurol. 1989;46(10):1121-3
